# Biosynthesis of Antibacterial Iron-Chelating Tropolones in *Aspergillus nidulans* as Response to Glycopeptide-Producing Streptomycetes

**DOI:** 10.3389/ffunb.2021.777474

**Published:** 2022-01-03

**Authors:** Jennifer Gerke, Anna M. Köhler, Jan-Peer Wennrich, Verena Große, Lulu Shao, Antje K. Heinrich, Helge B. Bode, Wanping Chen, Frank Surup, Gerhard H. Braus

**Affiliations:** ^1^Department of Moleuclar Microbiology and Genetics and Göttingen Center for Molecular Biosciences (GZMB), Georg-August-Universität Göttingen, Göttingen, Germany; ^2^Microbial Drugs Department, Helmholtz Centre for Infection Research (HZI), Braunschweig, Germany; ^3^German Centre for Infection Research (DZIF), Partner Site Hannover-Braunschweig, Braunschweig, Germany; ^4^Molecular Biotechnology, Goethe University Frankfurt, Frankfurt am Main, Germany; ^5^Department of Natural Products in Organismic Interactions, Max Planck Institute for Terrestrial Microbiology, Marburg, Germany

**Keywords:** *Aspergillus nidulans*, Streptomyces, glycopeptide antibiotics, structure elucidation, tropolones, fungal-bacterial co-cultivation

## Abstract

The soil microbiome comprises numerous filamentous fungi and bacteria that mutually react and challenge each other by the production of bioactive secondary metabolites. Herein, we show in liquid co-cultures that the presence of filamentous Streptomycetes producing antifungal glycopeptide antibiotics induces the production of the antibacterial and iron-chelating tropolones anhydrosepedonin (**1**) and antibiotic C (**2**) in the mold *Aspergillus nidulans*. Additionally, the biosynthesis of the related polyketide tripyrnidone (**5**) was induced, whose novel tricyclic scaffold we elucidated by NMR and HRESIMS data. The corresponding biosynthetic polyketide synthase-encoding gene cluster responsible for the production of these compounds was identified. The tropolones as well as tripyrnidone (**5**) are produced by genes that belong to the broad reservoir of the fungal genome for the synthesis of different secondary metabolites, which are usually silenced under standard laboratory conditions. These molecules might be part of the bacterium-fungus competition in the complex soil environment, with the bacterial glycopeptide antibiotic as specific environmental trigger for fungal induction of this cluster.

## Introduction

Soil is a complex and heterogenous ecosystem highly colonized by microorganisms (Karlovsky, 2008). The dynamic interactions developed by microorganisms in soil to compete for nutrients and space range from symbiotic to antagonistic. Thus, microorganisms have developed a chemical arsenal for signaling, defense and protection in multispecies communities (Linares et al., 2006; Scherlach et al., 2013; Granato et al., 2019; Gerke et al., 2020). These low-molecular-weight organic compounds are called secondary metabolites (SMs, also specialized metabolites or natural products) and are, unlike primary metabolites, not primarily involved in growth, development or reproduction of the producing organism although there are regulatory ties between secondary and primary metabolism (Keller, 2019).

SMs represent a significant source for medical relevant drugs (Watve et al., 2001). Two-thirds of antibiotics with natural origin that are valuable in medicine or agriculture are produced by Streptomyces species (Bentley et al., 2002; Chater, 2006). One of those antibiotics is the glycopeptide bleomycin. It is an antitumor agent and produced by a non-ribosomal peptide synthetase (NRPS) and a polyketide synthase (PKS) (Shen et al., 1999; Galm et al., 2008). Streptomyces are Gram-positive bacteria and form the largest genus of Actinobacteria (Euzéby, 1997). They live in soil as decomposers of plant material in competition with other soil-borne microorganisms such as fungi. Similar to filamentous fungi, Streptomyces exhibit a mycelium with aerial filaments that can bear more than 50 spherical airborne spores (Bentley et al., 2002; Chater, 2006; Chater et al., 2010).

The filamentous fungus *Aspergillus nidulans* lives in the soil where it forms a hyphal network (Riquelme et al., 2018; Gerke et al., 2020). It can produce a huge arsenal of SMs, the function of which is far from all described. The genes responsible for biosynthesis, transport and regulation of SMs are usually clustered in the fungal genome (Keller, 2019; Rokas et al., 2020). *A. nidulans* harbors more than 60 different gene clusters (Inglis et al., 2013). Until today and even though *A. nidulans* is a fungal research model organism since decades, <50% have been linked to SM products (Romsdahl and Wang, 2019). Identification of SMs often proves difficult because most of the biosynthetic gene clusters are silenced under standard laboratory conditions. Over the last years, various methods have been developed to activate these gene clusters (Brakhage and Schroeckh, 2011; Gerke and Braus, 2014; Zhang et al., 2019). Mimicking natural competition in the soil by co-cultivation of *A. nidulans* with *Streptomyces rapamycinicus* revealed that the physical interaction induced the expression of silent fungal biosynthetic gene clusters (Schroeckh et al., 2009).

Here, we show that not only the physical interaction, but also the presence of a bacterial metabolite can serve as signal for the induction of a biosynthetic gene cluster producing tropolones in *A. nidulans*. Co-cultivation of *A. nidulans* with the glycopeptide antibiotic-producing *Streptomyces mobaraensis* induced the biosynthesis of the tropolones anhydrosepedonin (**1**) and antibiotic C (**2**) which both inhibit bacterial growth. They are formed by the PKS-containing and 2,4-dihydroxy-3-methyl-6-(2-oxopropyl)benzaldehyde (DHMBA)-forming (**3**) *dba* (derivative of benzaldehyde) gene cluster in combination with the co-regulated, adjacent gene AN7893 (*troA*) encoding a non-heme Fe(II) dioxygenase. The addition of pure glycopeptide antibiotics such as bleomycin or phleomycin was sufficient to induce the fungal response. Further, the metabolites azanidulone (**4**) and tripyrnidone (**5**) were identified to be produced phleomycin-dependently by the *dba* cluster. Finally, the biosynthesis of the *dba*-dependent SMs was identified. These results confirm that SMs can serve as chemical weapons in a competition between *A. nidulans* and *S. mobaraensis*.

## Materials and Methods

### Strains and Culture Conditions

*Escherichia coli* DH5α and *Morganella morganii* SBK3 were cultivated in Lysogeny Broth (LB), containing 1% (w/v) bacto tryptone, 1% (w/v) NaCl and 0.5% (w/v) yeast extract, at 37°C. *A. nidulans* strains were cultivated in Minimal Medium (MM) [AspA (70 mM NaNO_3_, 11.2 mM KH_2_PO_4_, 7 mM KCl, pH 5.5), 1% (w/v) glucose, 0.1% (v/v) Hutner's trace elements] or in London medium (LM) [1% (w/v) glucose, 7 mM KCl, 2 mM MgSO_4_, 11.2 mM KH_2_PO_4_, 0.1% (v/v) Hutner's trace elements (Hill and Kafer, 2001) without iron sulfate, 10 μM FeCl_3_, 10 mM NaNO_3_, pH = 6.5] with 0.0001% (w/v) 4-aminobenzoic acid at 30 or 37°C. Two percentage (w/v) agar was added for cultivation in petri dishes. For induction of the *dba* gene cluster, 0.1 μg/ml bleomycin (VWR, 203408-10) or 1 μg/ml phleomycin (InvivoGen, ant-ph-5p) were added. All Aspergillus strains were constructed in AGB552 (Bayram et al., 2012) background. Streptomyces strains were cultivated in GYM medium (4 g/l glucose, 4 g/l yeast extract, 10 g/l malt extract, pH = 7.2). For solid GYM medium, 2% (w/v) agar and 2 g/l CaCO_3_ were added. For co-cultivation of Streptomyces strains with *A. nidulans*, 93 ml London medium were mixed with 32 ml GYM medium and inoculated with each 1.25^*^10^7^ spores of *Streptomyces* spp. and *A. nidulans* AGB552. The cultures were grown for 2 days at 120 rpm and 30°C. *S. mobaraenis* (DSM 40847) was provided by Deutsche Sammlung von Mikroorganismen und Zellkulturen GmbH (DSMZ). *Streptomyces coelicolor* and *Streptomyces cellulosae subsp. griseorubiginosus* were provided by Axel Zeeck. *M. morganii* SBK3 was provided by Günther Winkelmann.

### Plasmid and *A. nidulans* Strain Construction

For the construction of plasmids used for gene deletions in *A. nidulans*, pME4319 served as vector, which contains a recyclable phleomycin resistance cassette (Phle resistance gene from *Streptoalloteichus hindustanus* (*ble*) (Drocourt et al., 1990; Punt and van den Hondel, 1992; Hartmann et al., 2010; Liu et al., 2021). Sequences of all used primers in this study are listed in Supplementary Table 1. Approximately 1,500 bp of the 5' and 3' flanking regions of the gene of interest were inserted into the *Swa*I and *Pml*I sites of pME4319, respectively, by using the GeneArt™ Seamless Cloning and Assembly Enzyme Mix or the GeneArt™ Seamless Cloning and Assembly Kit (Thermo Fisher Scientific). The 5' and 3' flanking regions were amplified from *A. nidulans* genomic DNA with the primer pairs given in Supplementary Table 2. For transformation of *A. nidulans*, the deletion cassettes were excised from the corresponding plasmids with *Pme*I. Supplementary Table 3 includes all plasmids and Supplementary Table 4 all *A. nidulans* strains used in this study. *E. coli* and *A. nidulans* were transformed using the heat-shock method (Inoue et al., 1990) or the polyethylene glycol-mediated protoplast fusion method (Punt and van den Hondel, 1992), respectively. 5 μg/ml phleomycin was added to the medium to select for positive *A. nidulans* clones. The recyclable marker cassette in *A. nidulans* was removed by cultivation of the fungus on Minimal Medium containing 0.5% (w/v) glucose and 0.5% (w/v) xylose. Thereby, the xylose inducible promoter of the cassette activates expression of the ß-recombinase, whose gene product recognizes and cleaves off the marker cassette at its two flanking β*-six* recognition sites, leaving one β*-six* site (Hartmann et al., 2010; Liu et al., 2021).

### Quantitative Real-Time Polymerase Chain Reaction

Fungal mycelia were harvested, frozen in liquid nitrogen and ground with a table mill. RNA was isolated according to the instruction of the RNeasy® Plant Miniprep Kit (Qiagen). Approximately 0.8 μg of RNA was used for cDNA synthesis according to manufacturer's instructions of the QuantiTect® Reverse Transcription Kit (Qiagen). RT-PCR was performed with the MESA GREEN qPCR MasterMix Plus for SYBR Assay (Eurogentec) in a CFX Connect Real-Time System (BioRad). One microliter of 1:10 diluted cDNA was used per reaction. Two biological replicates with each three technical replicates were used. Primers are listed in Supplementary Table 5. Data were recorded with the CFX Manager software (BioRad). Expression of the housekeeping genes *h2A, gpdA*, and *rps15* were used for normalization.

### SM Extraction and LC-MS Analysis

For SM extraction from liquid cultures, mycelia were removed by filtration. The aqueous filtrates were adjusted to pH 5 and extracted with equivalent volumes of ethyl acetate. The organic solvent was evaporated, and the dried metabolites were stored at −20°C. For LC-MS analysis, the metabolites were reconstituted in acetonitrile/H_2_O (1:1) and analyzed with a Q Exactive™ Focus orbitrap mass spectrometer coupled to an UltiMate™ 3000 HPLC (Thermo Fisher Scientific) equipped with a DAD-3000 Diode Array Detector (Thermo Fisher Scientific) and a Corona™ Veo™ RS Charged Aerosol Detector (Thermo Fisher Scientific). Five microliter of each sample was injected on a HPLC column [Acclaim™ 120, C18, 5 μm, 120 Å, 4.6 × 100 mm (Thermo Fisher Scientific)] applying a linear acetonitrile/0.1% (v/v) formic acid in H_2_O/0.1% (v/v) formic acid gradient [from 5 to 95% (v/v) acetonitrile/0.1 formic acid in 20 min, plus additional 10 min with 95% (v/v) acetonitrile/0.1 formic acid] with a flow rate of 0.8 ml/min at 30°C. The measurements were performed in a mass range of 70–1,050 *m/z* in positive and negative mode. Data acquisition and analysis was performed with Thermo Scientific Xcalibur™ 4.1 (Thermo Fisher Scientific) and with FreeStyle™ 1.6 (Thermo Fisher Scientific) software.

For SM extraction of the fungal biomass, the mycelia were washed with water and dried. The mycelia were covered with 100 ml acetone and placed in a Sonorex™ Digital 10 P ultrasonic bath (Bandelin) for 30 min at 10% performance. The mycelia were removed by filtration and anhydrous magnesium sulfate was added to the organic filtrate to bind remaining water. After another filtering the organic solvent was evaporated as described before. For the HR-ESI-MS analysis, the metabolites were reconstituted in acetonitrile/H_2_O (1:1) and 2 μl was injected into an Agilent 1200 Infinity Series HPLC (Agilent Technologies, Böblingen, Germany) utilizing a C18 Acquity® UPLC BEH column (2.1 × 50 mm, 1.7 μm; Waters, Milford, MA/USA) connected to a maXis® ESI-TOF-MS (Bruker). HPLC parameters were set as follows: solvent A: H_2_O + 0.1% formic acid, solvent B: acetonitrile + 0.1% formic acid; gradient: 5% B (0.5 min), 5–100% (19.5 min), 100% (5 min), flowrate 0.6 ml/min, and DAD detection 190–600 nm.

### Instrumentation for Structure Elucidation of Anhydrosepedonin (1), Antibiotic C (2), and Tripyrnidone (5)

NMR spectra were recorded with an Avance III 700 spectrometer with a 5 mm TCI cryoprobe (^1^H 700 MHz, ^13^C 175 MHz) and an Avance III 500 spectrometer (^1^H 500 MHz, ^13^C 125 MHz; both Bruker, Billerica, MA/USA). NMR data were referenced to selected chemical shifts of acetone-*d*_6_ (^1^H: 2.05 ppm, ^13^C: 29.32 ppm), pyridine-*d*_5_ (^1^H: 7.22 ppm, ^13^C: 123.87 ppm) and DMSO-*d*_6_ (^1^H: 7.27 ppm, ^13^C: 77.00 ppm), respectively. Optical rotations were taken with a MCP 150 polarimeter (Anton Paar, Graz, Austria); UV spectra were taken with a UV-2450 UV/VIS spectrophotometer (Shimadzu, Kyoto, Japan); ECD spectra were collected with a JD 815 spectrophotometer (Jasco, Pfungstadt, Germany).

ESI-MS spectra were recorded with an UltiMate® 3000 Series uHPLC (Thermo Fisher Scientific, Waltman, MA/USA) utilizing a C18 Acquity® UPLC BEH column (2.1 × 50 mm, 1.7 μm; Waters, Milford, MA/USA) connected to an amaZon® speed ESI Iontrap MS (Bruker). HPLC parameters were set as follows: solvent A: H_2_O + 0.1% formic acid, solvent B: acetonitrile + 0.1% formic acid; gradient: 5% B (0.5 min), 5–100% (19.5 min), 100% (5 min), flowrate 0.6 mL/min, and DAD detection 190–600 nm. HR-ESI-MS spectra were obtained with an Agilent 1200 Infinity Series HPLC (Agilent Technologies, Böblingen, Germany; conditions same as for ESI MS spectra) connected to a maXis® ESI-TOF-MS (Bruker).

### Crude Extract Preparation for the Isolation of Anhydrosepedonin (1) and Antibiotic C (2)

Anhydrosepedonin (**1**) and antibiotic C (**2**) were isolated from crude extracts of *A. nidulans* strains: AGB535 (Δ*dbaF*) was used for the isolation of the mixture of anhydrosepedonin (**1**) and antibiotic C (**2**) as well as the isolation of pure antibiotic C (**2**), whereas anhydrosepedonin (**1**) was purified from AGB1430 (Δ*dbaB*). In total 5 L liquid LM supplemented with 10 μM FeCl_3_, 0.0001% (w/v) 4-aminobenzoic acid and 1 μg/ml phleomycin (InvivoGen, ant-ph-5p) was inoculated with 1 × 10^6^ spores/ml of either AGB535 or AGB1430 and incubated on a shaker for 2 days at 30°C. Mycelia were removed by filtration, the aqueous filtrates were adjusted to pH 5 and extracted with equivalent volumes of ethyl acetate. The organic solvent was evaporated, and the crude extracts were stored at −20°C.

### Isolation of Mixture of Anhydrosepedonin (1)/Antibiotic C (2)

98 mg crude extract was dissolved in 3 mL MeOH/DMSO (9:1) and subjected to preparative reverse phase HPLC (Pure C-850 FlashPrep, Büchi Labortechnik, Flawil, Switzerland). As stationary phase, Synergi Polar-RP column (250 × 50 mm, 10 μm, Phenomenex, Torrance, CA, USA) was used. The mobile phase consisted of deionized water (solvent A) and acetonitrile (solvent B). Purification of the crude extract was performed by using an isocratic elution of 5% aqueous ACN with 0.05% formic acid at a flow rate of 50 mL/min for 5 min, 5–100% solvent B in 60 min and finally isocratic elution at 100% solvent B for 10 min to afford the mixture of compound **1** and **2** (_tR_: 37.7–38.1 min, 12.1 mg).

### Isolation of Anhydrosepedonin (1)

220 mg crude extract of AGB1430 (Δ*dbaB*) was dissolved in 5 mL MeOH and subjected to preparative reverse phase HPLC (PLC 2250, Gilson, Middleton, WI, USA). As stationary phase, Synergi Polar-RP column (250 × 50 mm, 10 μm, Phenomenex, Torrance, CA, USA) was used. The mobile phase consisted of deionized water (solvent A) and acetonitrile (solvent B). Purification of the crude extract was performed by using an isocratic elution of 15% aqueous ACN with 0.05% formic acid at a flow rate of 50 mL/min for 5 min, 15–50% solvent B in 50 min, 50–100% solvent B in 10 min, and finally isocratic elution at 100% solvent B for 10 min to afford compound **1** (*t*_R_: 46.0–46.5 min, 12.7 mg).

### Isolation of Antibiotic C (2)

148.6 mg crude extract of AGB535 (Δ*dbaF*) was dissolved in 6 mL MeOH, divided into 4 equal parts and subjected to preparative reverse phase HPLC (Pure C-850 FlashPrep, Büchi Labortechnik, Flawil, Switzerland). As stationary phase, Luna C18(2) column (250 × 21.2 mm, 5 μm, Phenomenex, Torrance, CA, USA) was used. The mobile phase consisted of deionized water (solvent A) and acetonitrile (solvent B). Purification of the crude extract was performed by using an isocratic elution of 15% aqueous ACN with 0.05% formic acid at a flow rate of 20 mL/min for 5 min, 15–45% solvent B in 32 min, 50–100% solvent B in 3 min, and finally isocratic elution at 100% solvent B for 10 min to afford compound **2** (*t*_R_: 32.0–32.3 min, 3.6 mg).

### Isolation of 7-Hydroxy-3,7-Dimethyl-6H-2-Benzopyran-6,8(7H)-Dione (Azanidulone, 4)

Ten liter liquid MM was inoculated with 10^9^ spores of strain AGB527 and grown at 37°C for 36 h. Mycelia were removed by filtering with Miracloth and the pH of the culture filtrate was adjusted to 5. The culture filtrate was extracted twice with an equivalent amount of ethyl acetate and dried to yield the crude extract. The crude extract was separated by preparative HPLC (Instrumentelle Analytik Goebel GmbH: HPLC pump RAININ Dynamax SD-1, HPLC detector RAININ Dynamyx UV-1, column Nucleodur 100-5 C18 ec, 250 × 20 mm, solvent system: A = H_2_O, B = acetonitrile) under gradient conditions (20% B to 100% B in 20 min). Detection was carried out at 230 nm. The isolation yielded 7.7 mg 7-hydroxy-3,7-dimethyl-6H-2-benzopyran-6,8(7H)-dione that we named azanidulone. ^1^H-NMR spectra were recorded on a *Varian* Mercury-Vx 300 (300 MHz) spectrometer. ^13^C-NMR spectra were recorded on a *Varian* Inova-500 spectrometer (125.7 MHz). ESI-MS data were acquired using a *Finnigan* LC-Q mass spectrometer.

### Isolation of Tripyrnidone (5)

Four liter liquid LM with 1 μg/ml phleomycin was inoculated with 10^9^ spores of strain AGB1418 and grown at 30°C for 2 days. Mycelia were removed and the pH of the culture filtrate was adjusted to 5. The culture filtrate was extracted twice with an equivalent amount of ethyl acetate and dried to yield 515.9 mg crude extract. The crude extract was dissolved in 10 mL MeOH and subjected to preparative reverse phase HPLC (PLC 2020, Gilson, Middleton, WI, USA). As stationary phase, VP Nucleodur 100-5 C18 ec column (250 × 20 mm, 7 μm, Macherey-Nagel, Düren, Germany) was used. The mobile phase consisted of deionized water (solvent A) and acetonitrile (solvent B). Purification of the crude extract was performed by using a linear gradient elution of 10–30% aqueous ACN with 0.05% formic acid at a flow rate of 20 mL/min for 35 min, 30–50% solvent B in 10 min, 50–100% solvent B in 5 min, and finally isocratic elution at 100% solvent B for 10 min to afford compound **5** (*t*_R_: 29–29.8 min, 3 mg).

### Spectroscopic Data

#### (1) Anhydrosepedonin

C_11_H_10_O_4_, yellow solid, ESI-MS: *m/z* 206.9 [M + H]^+^, 204.9 [M – H]^−^. HRESIMS: *m/z* 207.0651 [M + H]^+^ (calcd. for C_11_H_10_O_4_ 207.0657). UV (AcCN/H_2_O/0.1%FA) λ_max_ 221 (sh), 250, 288, 381 nm. ^1^H-NMR (500 MHz, DMSO-*d*_6_): δ_*H*_ 6.76 (s, 1H, 7-H), 6.60 (s, 1H, 3-H), 5.73 (s, 1H, 8-H), 5.00 (s, 1H, 11-H), 1.93 (s, 3H, 10-H). ^13^C-NMR (125 MHz, DMSO-*d*_6_): δ_*C*_ 19.1 (CH_3_, C-10), 64.5 (CH_2_, C-11), 105.2 (CH, C-8), 111.2 (CH, C-7), 112.4 (CH, C-3), 112.7 (C, C-5), 140.9 (C, C-4), 161.2 (C, C-9), 161.6 (C, C-6), 165.8 (C, C-2), 169.8 (C, C-1).

#### (2) Antibiotic C

C_11_H_10_O_5_, yellow solid, ESI-MS: *m/z* 222.9 [M + H]^+^, 220.8 [M – H]^−^. HRESIMS: m/z 223.0597 [M + H]^+^ (calcd. For C_11_H_10_O_5_ 223.0607). UV (AcCN/H_2_O/0.1%FA) λ_max_ 195, 223, 271, 305 (sh), 365, 385 nm. ^1^H-NMR (500 MHz, DMSO-*d*_6_): δ_*H*_6.59 (s, 1H, 3-H), 5.70 (s, 1H, 8-H), 5.14 (s, 1H, 11-H), 1.91 (s, 3H, 10-H). ^13^C-NMR (125 MHz, DMSO-*d*_6_): δ_*C*_ 19.1 (CH_3_, C-10), 64.6 (CH_2_, C-11), 104.2 (CH_2_, C-8), 113.2 (CH, C-3), 114.1 (C, C-5), 132.9 (C, C-4), 146.5 (C, C-2), 154.3 (C, C-1), 155.1 (C, C-6), 157.8 (C, C-9), 160.9 (C, C-7).

#### (4) Azanidulone [(7S)-7-Hydroxy-3,7-Dimethyl-6H-2-Benzopyran-6,8(7H)-Dione]

C_11_H_10_O_4_, yellow solid, ESI-MS: *m/z* (%) 229.1 [M + Na]^+^, 207.1 [M + H]^+^. ^1^H-NMR (300 MHz, CD_3_OD): δ_*H*_ 1.46 (s, 3H, 10-H), 2.19 (s, 3H, 9-H), 5.44 (s, 1H, 5-H), 6.34 (s, 1H, 4-H), 8.01 (s, 1H, 1-H). ^13^C-NMR (500 MHz, CD_3_OD): δ_*C*_ 19.2 (CH_3_, C-11), 28.0 (CH_3_, C-12), 84.5 (C, C-7), 106.1 (CH, C-5), 109.8 (CH, C-4), 117.0 (C, C-9), 146.2 (C, C-10), 154.9 (CH, C-1), 161.5 (C, C-3), 198.6 (C, C-8), 198.9 (C, C-6). UV λ_max_ (acetonitrile/H_2_O/formic acid)/nm: 228, 338. ^1^H-NMR data are in agreement with reported data (Marsini et al., 2006), ^13^C-NMR data are in agreement with reported data (Zhang et al., 2016).

#### (5) Tripyrnidone

C_17_H_16_O_7_: HRESIMS *m/z* 355.0777 [M + Na]^+^ (calcd. for C_17_H_16_O_7_Na, 355.0794), m/z 333.0961 [M + H]^+^ (calcd for C_17_H_17_O_7_, 333.0974), *m/z* 315.0857 [M − H_2_O + H]^+^ (calcd. for C_17_H_15_O_6_, 315.0869), *m/z* 331.0811 [M − H]^−^ (calcd. for C_17_H_15_O_7_, 331.0818). UV (AcCN/H_2_O/0.1%FA) λ_max_ 196, 217, 250 (sh), 292, 338 nm. ^1^H NMR (500 MHz, DMSO-*d*_6_): see Table 1; ^13^C NMR (125 MHz, acetone-*d*_6_): see Table 1; Rt 7.47 min.

**Table 1:** NMR data of tripyrnidone (5) in DMSO-*d*_6_ (^1^H 500 MHz, ^13^C 125 MHz).

	**5 main isomer**		**5 minor isomer**	
	****δ_C_**, mult**.	****δ_H_**, mult**.	****δ_C_**, mult**.	****δ_H_**, mult**.
1	29.5, CH_3_	2.19, s	29.1, CH_3_	2.16, s
2	204.4, C		204.6, C	
3	46.3, CH_2_	3.83, d (16.5)	46.9, CH_2_	3.74, d (15.8)
		3.76, d (16.5)		3.59, d (15.8)
4	147.8, C		146.5, C	
5	124.0, CH	5.92, s	124.4, CH	5.89, s
6	198.5, C		195.2, C	
7	78.9, C		75.3, C	
8	100.0, C	OH: 7.35, br s	100.4, C	OH: 7.88, s
9	126.3, C		126.5, C	
10	121.2, CH	6.89, s	119.6, CH	6.83, s
11	98.4, C		98.0, C	
12	163.2, C		163.0, C	
13	100.1, CH	6.34, m	99.9, CH	6.34, m
14	165.1, C		164.4, C	
15	19.8, CH_3_	2.28, br s	19.7, CH_3_	2.27, br s
16	160.8, C		160.9, C	
17	23.1, CH_3_	1.25, s	14.2, CH_3_	1.36, s

Data for all identified compounds are shown in Supplementary Figures 2–11 and Supplementary Tables 6–8.

### Bioassay for Detection of Siderophores With Keto-Hydroxy Bidentate Ligands

The bioassay for detection of siderophores with keto-hydroxy bidentate ligands was performed as described previously (Thieken and Winkelmann, 1993). Plates with solid LB medium (containing agar agar, SERVA, 9002-18-0) with or without 600 μM 2,2'-dipyridyl were inoculated with fresh *M. morganii* SBK3 over-night cultures. SMs were extracted from 125 ml liquid cultures of *A. nidulans* AGB552 and Δ*dbaI* as described above and dissolved in 100 μl methanol. Filter discs were coated with 10 μl metabolite extracts diluted 1:1 with methanol and placed on the agar. Ten microliter pure methanol served as control. Plates were cultivated for 3 days at 37°C in darkness. Growth and inhibition zones were detected and measured.

### Bioactivity Assays

Bioactivity assays were performed to analyze the direct growth inhibiting effect of the SM extracts from *A. nidulans* on Streptomyces. 2 × 10^6^ spores of *S. mobaraensis, S. coelicolor*, and *S. cellulosae* were distributed on whole agar plates containing 40 ml GYM medium. The SM extract of fungal reference strain AGB552 and Δ*dbaI* was dissolved in 80 μl methanol and 10 μl were spotted on a filter disk which was incubated with the inoculated plates for 2 days at 30°C. To test the direct effect of anhydrosepedonin and antibiotic C these metabolites were isolated from the fungal extracts by preparative HPLC. The anhydrosepedonin/antibiotic C mixture as well as separately isolated anhydrosepedonin (**1**) and antibiotic C (**2**) were dissolved in methanol to a final concentration of 2 mg/ml. 12.5 μl of the extract was spotted on filter disks, which were incubated with the Streptomyces inoculated GYM plates for 2 days at 30°C. Methanol served as control.

Minimum inhibitory concentrations (MIC) were determined as described previously (Becker et al., 2021). The following fungal and bacterial organisms were tested: Bacteria: *Bacillus subtilis* (DSM10), *Staphylococcus aureus* (DSM346), *Acinetobacter baumanii* (DSM30008), *Chromobacterium violaceum* (DSM30191), *E. coli* (DSM1116), *Pseudomonas aeruginosa* (PA14); mycobacteria: *Mycolicibacterium smegmatis* (ATCC700084); fungi: *Candida albicans* (DSM1665), *Schizosaccharomyces pombe* (DSM70572)*, Mucor hiemalis* (DSM2656), *Pichia anomala* (DSM6766), *Rhodotorula glutinis* (DSM10134). The cytotoxicity assay against mouse fibroblast cell line L929, human cervical cancer cell line KB 3.1, human epidermoid carcinoma cell line A431, human lung carcinoma cell line A549, human prostate carcinoma cell line PC-3, human ovarian adenocarcinoma cell line SKOV-3 as well as human breast adenocarcinoma cell line MCF-7 was performed as described by (Becker et al., 2021).

## Results

### Co-cultivation of *A. nidulans* With Glycopeptide-Forming *S. mobaraensis* Activates Production of Secondary Metabolites

To unravel the chemical response of *A. nidulans* toward different Streptomyces species, *A. nidulans* AGB552 was co-cultivated with *S. mobaraensis, S. coelicolor* or *S. cellulosae* on solid and in liquid medium at 30°C. The three Streptomyces strains are producers of different kinds of antibiotics (Höfs et al., 2000; Bentley et al., 2002; Komaki and Harayama, 2006).

**Figure 1:**
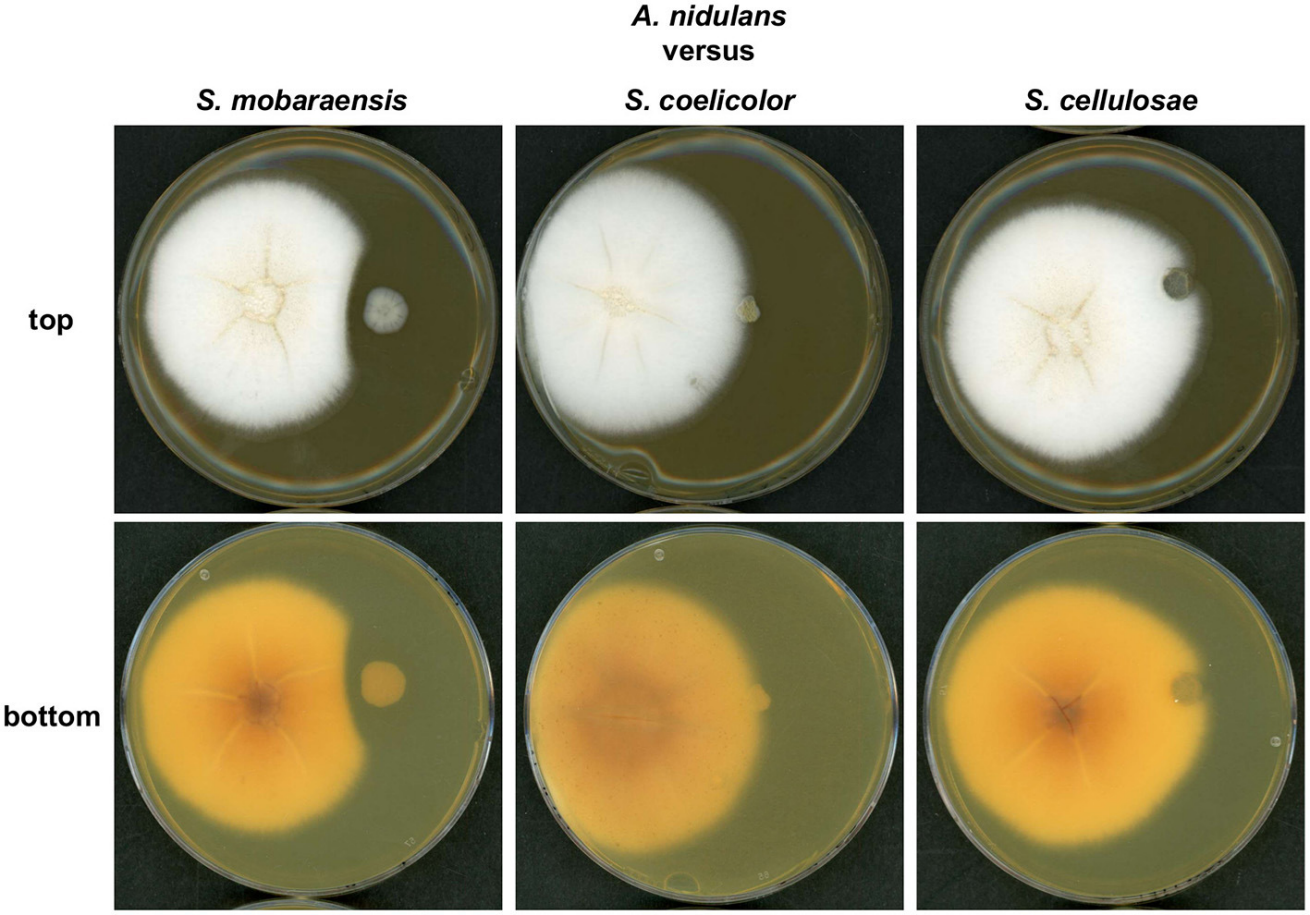
A growth inhibition zone is formed between colonies of *S. mobaraensis* and *A. nidulans* during co-cultivation. Confrontation assay of *A. nidulans* strain AGB552 with different Streptomycete strains (*S. mobaraensis, S. coelicolor* and *S. cellulosae)*. 10^4^ spores of *A. nidulans* (left colony) were point inoculated with 3 cm distance to 10^4^ spores of Streptomycete strains (right colony) on GYM medium and incubated at 30°C for 6 days.

First, solid GYM medium was inoculated with *A. nidulans* and one of the Streptomyces strains. After 6 days of growth an inhibition zone was visible between the colonies of *S. mobaraensis* and *A. nidulans* (Figure 1). No inhibition zone was observed when *S. coelicolor* or *S. cellulosae* were co-cultivated with *A. nidulans*. *S. mobaraensis* is a known producer of the glycopeptide antibiotic bleomycin, which is active against Aspergilli (Austin et al., 1990; Moore et al., 2003).

**Figure 2:**
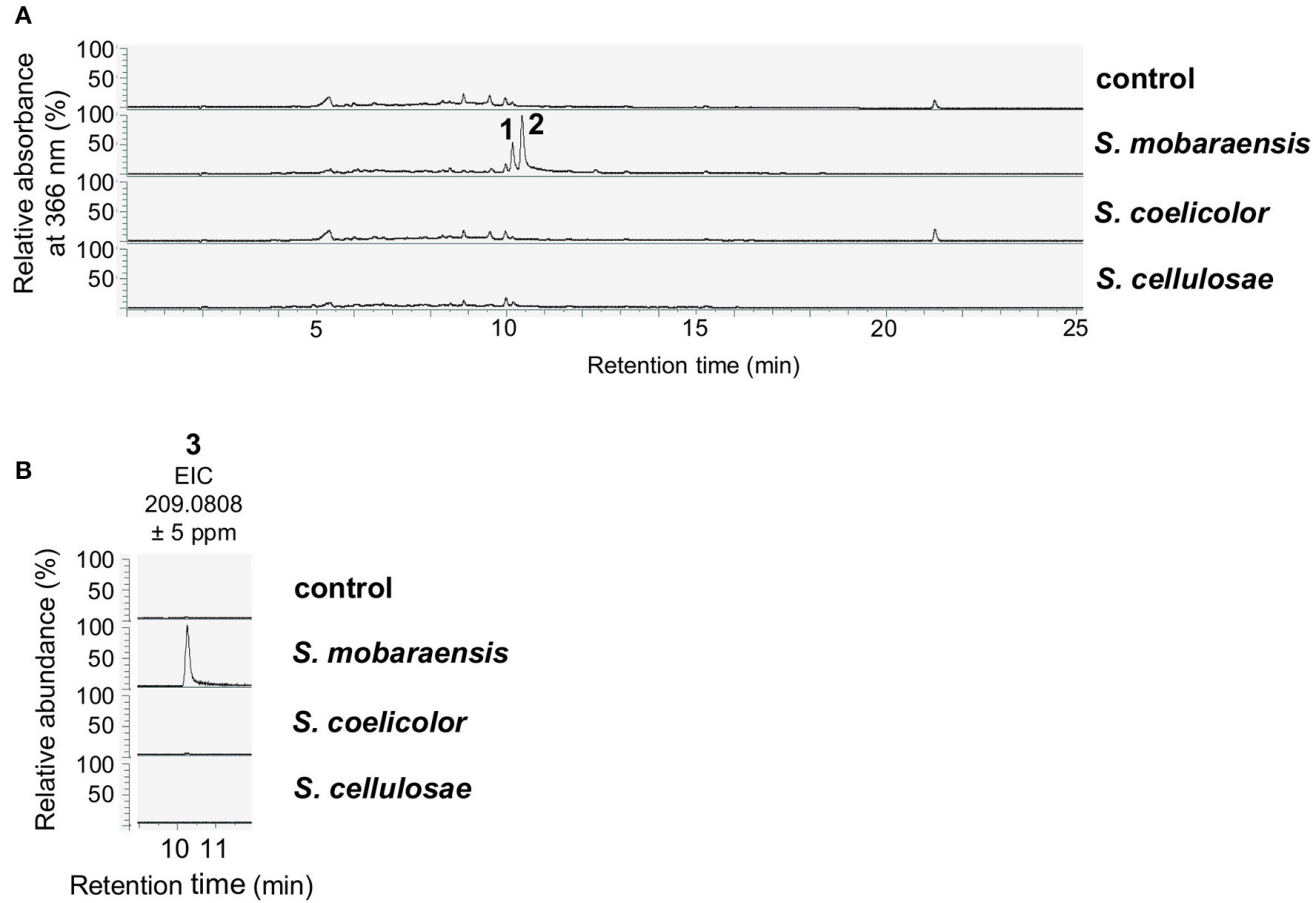
Co-cultivation of *A. nidulans* with *S. mobaraensis* induces the production of anhydrosepedonin (**1**), antibiotic C (**2**) and 2,4-dihydroxy-3-methyl-6-(2-oxopropyl)benzaldehyde (DHMBA, **3**). **(A)** *A. nidulans* reference strain AGB552 (rf) was co-cultivated with *S. mobaraensis, S. coelicolor* and *S. cellulosae* in liquid medium for 2 days. SMs were extracted from medium and analyzed with LC-MS/MS equipped with a DAD. The relative absorbance at 366 nm is shown. As control, only *A. nidulans* was cultivated. **(B)** Extracted ion chromatograms (EIC) for DHMBA (**3**).

Next, SMs from liquid co-cultivation experiments were extracted to analyze whether *A. nidulans* produces specific molecules as a chemical response to *S. mobaraensis*, which might induce bacterial inhibition. After 2 days of cultivation, mycelia were removed and the metabolites in the filtrate were extracted with ethyl acetate and analyzed in LC-MS/MS equipped with a diode array detector (DAD). The HPLC chromatogram at 366 nm revealed two peaks in the sample extracted from the *S. mobaraensis* co-culture that were not present in the other co-cultures (Figure 2).

### Glycopeptide Antibiotics Bleomycin and Phleomycin Are the Bacterial Signal for the Induction of Tropolone Production in *A. nidulans*

**Figure 3:**
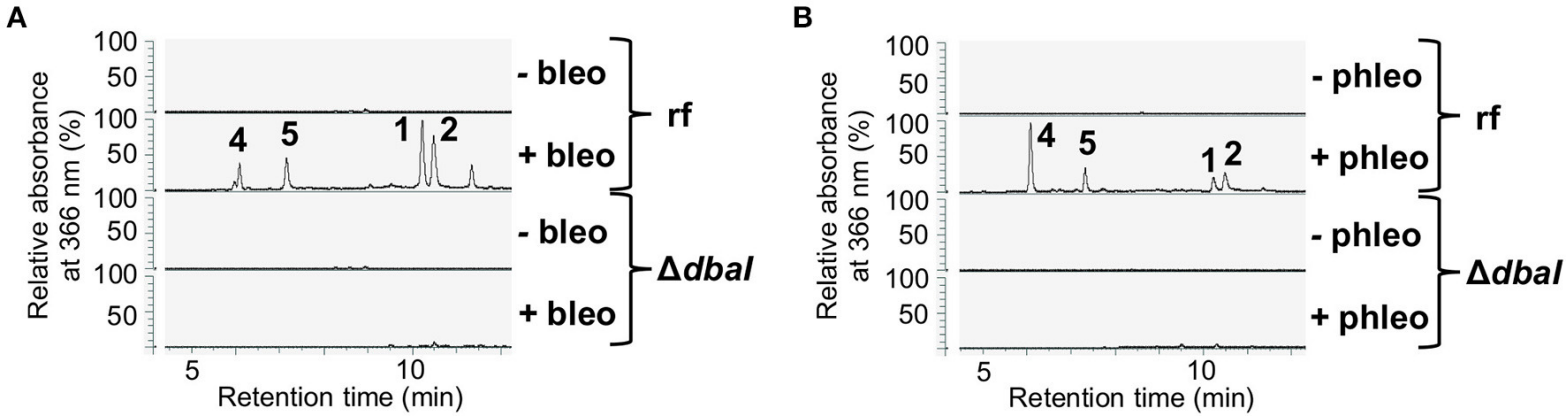
The presence of the glycopeptide antibiotics bleomycin and phleomycin induces the production of the *dbaI*-dependent metabolites **1**, **2**, **4**, and **5**. *A. nidulans* reference strain AGB552 (rf) was cultivated in liquid medium with or without the presence of 0.1 μg/ml bleomycin **(A)** or 1 μg/ml phleomycin **(B)** for 2 days. SMs were extracted from medium and analyzed with LC-MS/MS equipped with a DAD. The relative absorbance at 366 nm is shown.

*S. mobaraensis* is a producer of the glycopeptide antibiotic bleomycin, which shows antifungal activity against Aspergilli and other ascomycetes (Austin et al., 1990; Moore et al., 2003). A growth test of *A. nidulans* AGB552 on medium supplemented with different concentrations of bleomycin revealed that *A. nidulans* grows on low concentrations of 0.1 μg/ml bleomycin (Supplementary Figure 1). To unravel the bacterial signal for fungal production of the tropolones, *A. nidulans* AGB552 was cultivated in liquid medium supplemented with 0.1 μg/ml bleomycin. After metabolite extraction, the chromatogram revealed the production of several metabolites that were not present in extracts from medium without bleomycin (Figure 3A). Phleomycin is another glycopeptide antibiotic produced by Streptomyces and closely related to bleomycin. According to the literature, *A. nidulans* cannot grow at phleomycin concentrations of c ≥ 10 μg/ml (Punt and van den Hondel, 1992). A growth test of *A. nidulans* AGB552 on medium supplemented with phleomycin revealed fungal growth at 1 μg/ml phleomycin (Supplementary Figure 1A). Cultivation of *A. nidulans* AGB552 in liquid medium supplemented with 1 μg/ml phleomycin and subsequent metabolite analysis showed the production of metabolites **1** and **2** as well as the two new peaks **4** and **5** (Figure 3B) that were also detected in cultures induced by bleomycin (Figure 3A; Supplementary Table 6).

**Figure 4:**
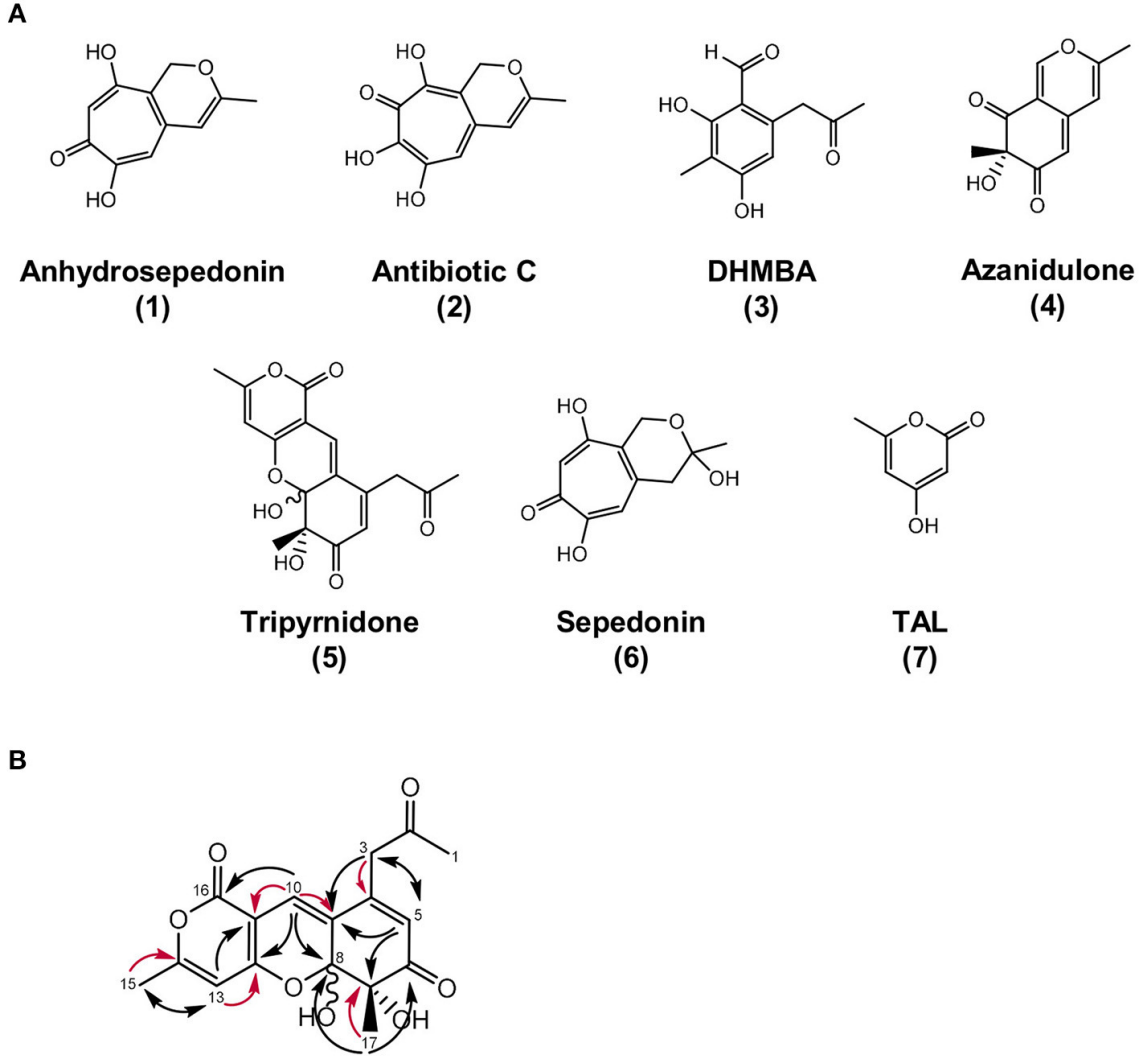
Overview of identified metabolites and their structures. **(A)** Chemical structures of anhydrosepedonin (**1**), antibiotic C (**2**), DHMBA (**3**), azanidulone (**4**), tripyrnidone (**5**), sepedonin (**6**) and TAL, (**7**). The stereochemistry of **6** is not known. **(B)** Selected 1,1 ADEQUATE (red arrows) and ^1^H,^13^C HMBC (black arrows) correlations of compound **5**.

Metabolites **1** and **2** were isolated by preparative HPLC and elucidated as the tropolones anhydrosepedonin (**1**) and antibiotic C (**2**) (Figure 4A) by NMR, HRESIMS and UV/VIS data. In addition, DHMBA (**3**) was identified by HRESIMS and UV/VIS spectra in small amounts from the *S. mobaraensis* co-culture. DHMBA (**3**) was identified earlier as PKS-derived metabolite in *A. nidulans* (Ahuja et al., 2012; Gerke et al., 2012). Anhydrosepedonin (**1**) has been isolated earlier from the fungus *Sepedonium chrysospermum* (Wright et al., 1970) and antibiotic C (**2**) from *A. nidulans* strain IMI238850 (McDonald et al., 1983), but no full set of NMR data has been published. The corresponding biosynthetic gene clusters for both compounds have not been identified, either. We conclude that the three identified compounds are produced by *A. nidulans* in co-culture with *S. mobaraensis*.

Compound **4** was isolated by preparative HPLC and identified as the azaphilone 7-hydroxy-3,7-dimethyl-6H-2-benzopyran-6,8(7H)-dione that we named azanidulone (Figure 4). It has been isolated before from the ascomycete *Daldinia concentrica* (Buchanan et al., 1995) and the ascomycete *Nigrospora* sp. YE3033 (Zhang et al., 2016). A 7*S* configuration was assigned due to a negative CE observed at ca. 355 nm in the CD spectrum of **4** (Supplementary Figure 7) (Clark et al., 2008).

Taken together, the glycopeptide antibiotics bleomycin and phleomycin, which are naturally produced by Streptomyces, induce the production of the tropolones anhydrosepedonin (**1**) and antibiotic C (**2**) as well as of DHMBA (**3**), azanidulone (**4**) and **5** in *A. nidulans*.

### Structure Elucidation of the Novel Compound Tripyrnidone (5)

Compound **5** was isolated by preparative HPLC as a red oil. Its UV/VIS spectrum with maxima at 292 and 389 nm indicated an extensive π-system. The molecular formula C_17_H_16_O_7_ was obtained from the peak at *m/z* 333.0972 in the HRESIMS spectrum, specifying 10 units of unsaturation. The ^1^H and ^13^C NMR spectra of **5** in DMSO-*d*_6_ contained two sets of resonances with a ratio of about 4:3, signifying spontaneous interconversion. Structure elucidation is described with data of the main isomer as follows: The proton and HSQC spectra of **5** indicated the presence of three methyls (δ_C_/δ_H_ 29.5/2.19, 23.1/1.25, 19.8/2.28), one methylene (δ_C_/δ_H_ 46.3/3.83 and 3.76) and three olefinic methines (δ_C_/δ_H_ 124.0/5.92, 121.2/6.89, 100.1/6.34). Additionally, the carbon NMR spectrum showed signals for two ketones (δ_*C*_ 204.4, 198.5) and eight additional (δ_C_ 165.1, 163.2, 160.8, 147.8, 126.3, 100.0, 98.4, 78.9) carbon atoms devoid bound protons. Since **5** has a ratio of H/C atoms of <1, the crowding of HMBC correlations from just a few protons complicated the structure elucidation process. Consequently, an ADEQUTE NMR spectrum was measured for the distinction between ^2^*J*_C, H_ and ^3^*J*_C, H_ correlations. Foremost, the assignment of two-bond correlations from 3-H to C−4, from 10–H to C−9 and C−11, from 13–H to C−12 and C−14, from 15–H_3_ to C−14 and from 17–H_3_ to C−7 facilitated the assignment. Subsequently, observed ^3^*J* correlations from the HMBC NMR spectrum assembled the planar structure on **5** (Figure 4B). The largest difference in chemical shift between the isomers was observed for methyl group C−17 (δ_C_ 14.2 vs. 23.1), suggesting that the isomers differ in this region of the molecule. Since all HMBC correlations are analogous for both isomers, a stereo chemical difference was expected. The stereochemistry of C−7 is immutable and can be deduced from the absolute configuration of the biosynthetically related azanidulone (**4**) as *7S*, but the neighboring hemiketal C−8 is metabolically unstable and prone to epimerize. Since a ROESY correlation between 8–OH and 17–H_3_ was observed for the minor isomer, 7*S*, 8*S* and 7*S*,8*R* absolute configurations were assigned for the minor and for the major epimer, respectively.

Compound **5** is a novel substance with a novel tricyclic scaffold that we named tripyrnidone. It showed no bioactivity in our test assays against bacteria or fungi and no cytotoxicity against the tested cell lines.

### Tropolones Are Biosynthesized by the Non-reducing PKS DbaI

DHMBA (**3**) was shown to be the direct product of the non-reducing PKS DbaI in *A. nidulans* (Ahuja et al., 2012; Gerke et al., 2012). We deleted *dbaI* in AGB552 background and analyzed the SM profile with LC-MS/MS with and without the addition of bleomycin and phleomycin (Figure 3). The deletion of *dbaI* abolished the production of DHMBA (**3**) as well as of the SMs **1**, **2, 4**, and **5**. It can be concluded that the tropolones as well as the compounds **4** and **5** are produced by the PKS DbaI and are most likely biosynthesized from DHMBA by other enzymes.

### Phleomycin Induces the Expression of the DHMBA-Producing *dba* Gene Cluster

**Figure 5:**
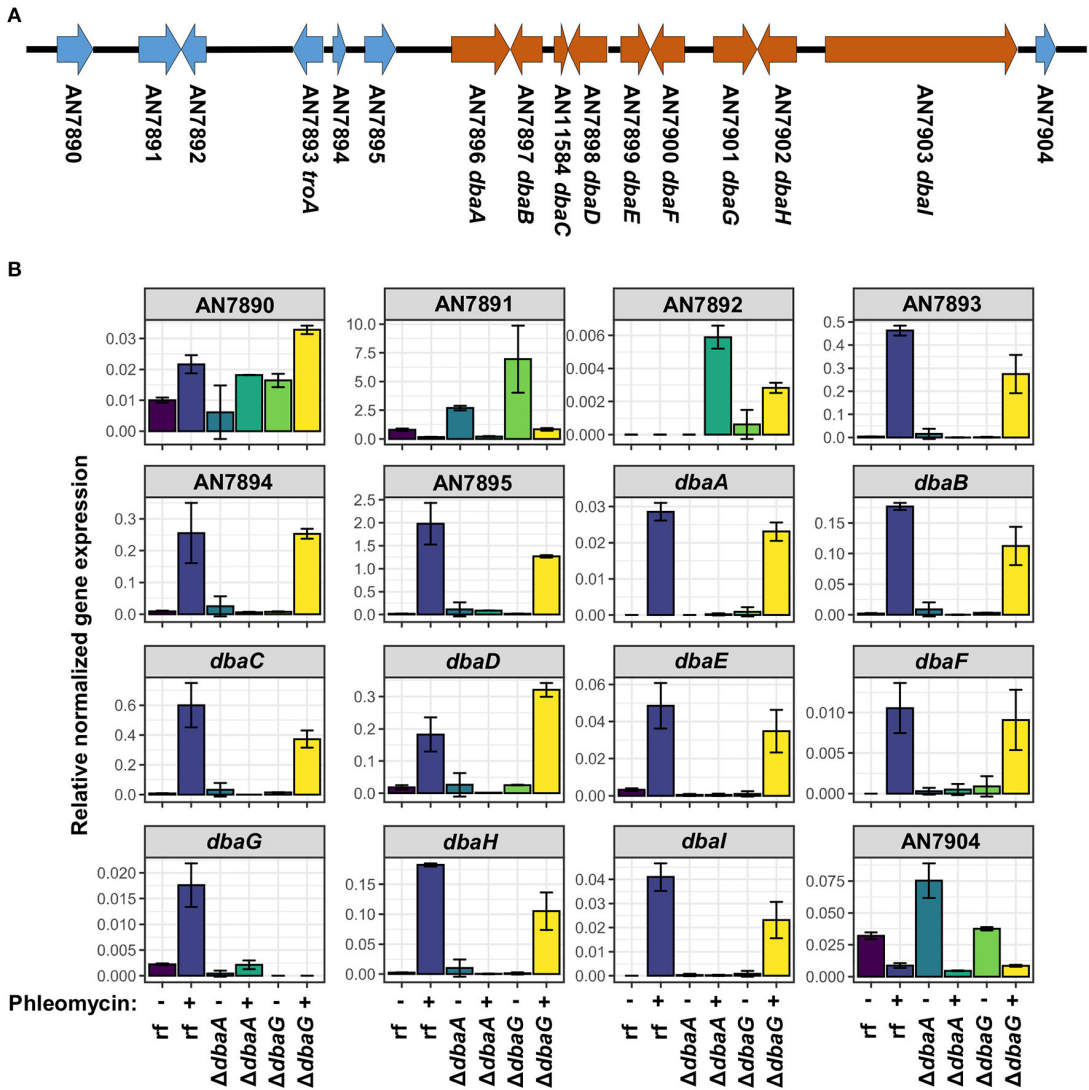
Expression of genes from AN7893 (*troA*) to AN7903 (*dbaI*) is induced in presence of phleomycin. **(A)** Scheme of *dba* gene cluster (orange) and neighbored genes (blue) on chromosome II of *A. nidulans*. **(B)** Relative gene expression levels of AN7890-AN7904 in reference strain AGB552 (rf), Δ*dbaA* and Δ*dbaG* after cultivation with (+) and without (–) the addition of 1 μg/ml phleomycin. Expression of the housekeeping genes *h2A, gpdA* and *rps15* were used for normalization. Error bars represent the standard error of two biological replicates.

In fungi, biosynthetic genes for a specific SM are usually clustered in the genome (Keller, 2019; Rokas et al., 2020). The gene encoding the DHMBA-producing PKS DbaI is embedded within the *dba* gene cluster on chromosome II of *A. nidulans* (Gerke et al., 2012) (Figure 5A). The cluster spans 9 genes, *dbaA*-*dbaI*, and encodes two putative transcription factors DbaA and DbaG. Overexpression of *dbaA* upregulates all genes of the cluster, which is usually silenced under standard laboratory conditions (Gerke et al., 2012). To understand the influence of glycopeptide antibiotics on *dba* gene cluster expression, the relative gene expressions of the *dba* cluster genes were analyzed with quantitative real-time polymerase chain reaction (qPCR). The neighbored genes AN7890-AN7895 and AN7904 were included in the analysis. The genes AN7893-7895 were shown earlier to be co-regulated with the *dba* cluster under certain conditions (Nahlik et al., 2010; Gerke et al., 2012; Bohnert et al., 2014; Oiartzabal-Arano et al., 2015). The *A. nidulans* reference strain AGB552 (rf) and the two transcription factor deletion strains Δ*dbaA* and Δ*dbaG* were cultivated with and without phleomycin. The relative gene expression pattern showed that all 9 *dba* genes as well as AN7893-AN7895 were upregulated in the reference strain when phleomycin was added (Figure 5B). In contrast, expression of AN7893-*dbaI* was not increased in phleomycin-containing medium when *dbaA* was deleted, proving that DbaA is the specific transcriptional activator for the gene cluster. The putative transcription factor DbaG is not involved in *dba* gene regulation but showed a repressive function on expression of the uncharacterized AN7891 gene, which encodes a β-1,4-endoglucanase, in medium without phleomycin. In summary, the presence of 1 μg/ml phleomycin induces the expression of the *dba* genes plus AN7893-AN7895. To test what is the lowest phleomycin concentration for the gene cluster induction, we performed qPCR with RNA extracted from cultures containing 0.01 to 1 μg/ml phleomycin. The analysis revealed expression of *dbaI* at 0.5 and 1 μg/ml phleomycin, but lower concentrations are rather insufficient (Supplementary Figure 1B).

### Addition of the Glycopeptide Antibiotics Bleomycin and Phleomycin Induces the Production of Different Metabolites of the *dba* Biosynthetic Gene Cluster

**Figure 6:**
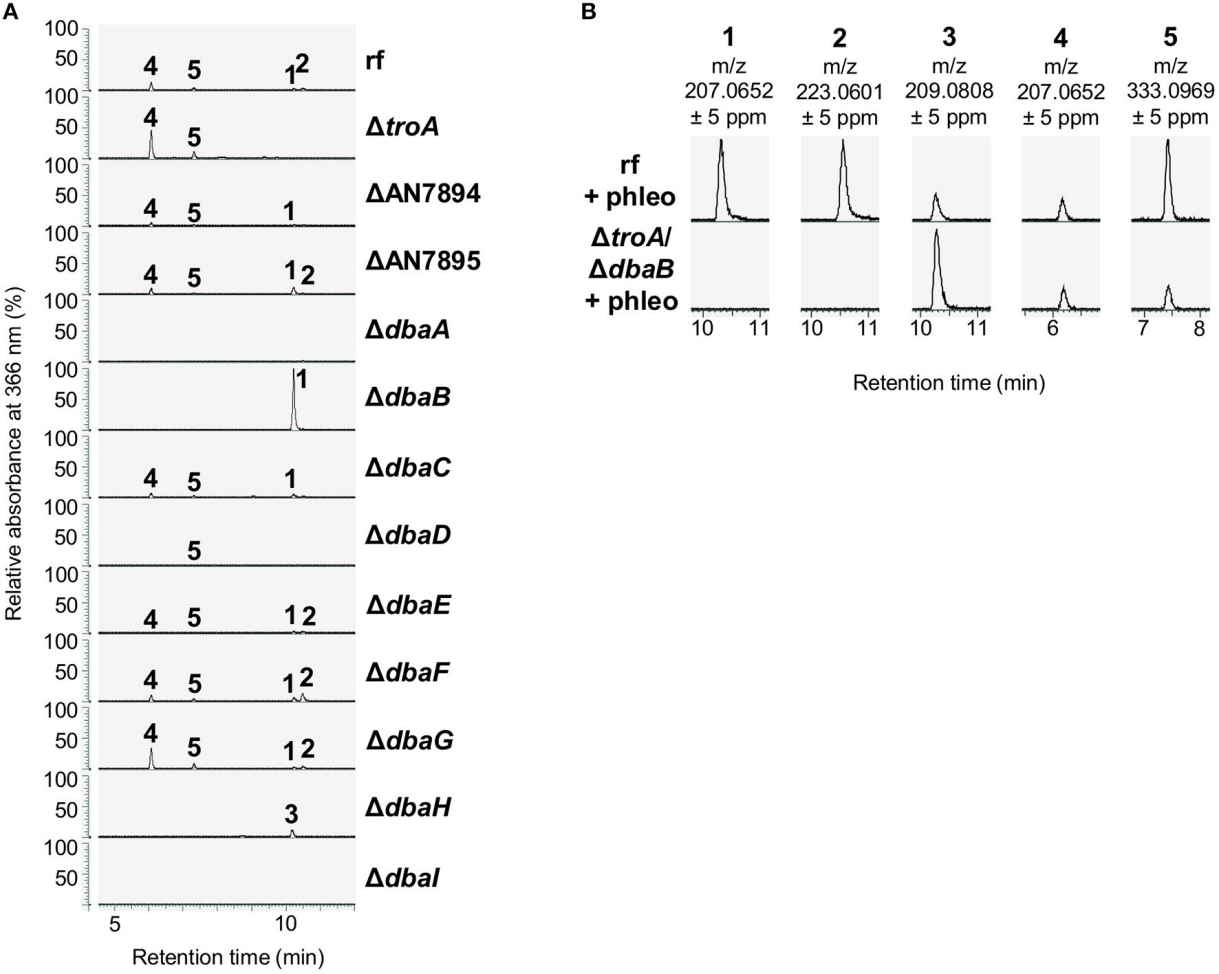
The *dba*/*troA* cluster produces the tropolones anhydrosepedonin and antibiotic C. **(A)** The indicated strains were cultivated in liquid medium supplemented with 1 μg/ml phleomycin for 2 days. Ethyl acetate extracts of culture filtrates were analyzed with LC-MS/MS equipped with a DAD. The relative absorbance at 366 nm is shown. (**1**) anhydrosepedonin, (**2**) antibiotic C, (**3**) DHMBA, (**4**) azanidulone, (**5**) tripyrnidone. **(B)** Extracted ion chromatograms of **1**–**5** in positive ionization mode. Extracted ion chromatograms of all compounds are shown in Supplementary Figure 12.

Compounds **1–5** are polyketides. The structures as well as the abolished production in the PKS deletion strain Δ*dbaI* suggest that the compounds **1**, **2**, **4**, and **5** are synthesized from the DbaI-product DHMBA (**3**) (Gerke et al., 2012) by other co-regulated enzymes. Therefore, all genes from AN7893-*dbaI* were deleted in *A. nidulans* AGB552 background. A SM analysis with LC-MS/MS after cultivation in medium with phleomycin revealed that without the transcriptional activator DbaA and without the PKS DbaI the compounds **1–5** were not produced (Figure 6A; Supplementary Figure 12). Deletion of the transcription factor gene *dbaG* as well as AN7895, *dbaE* and *dbaF* had no impact on compound production. In the strain without the transporter gene *dbaD*, the compounds **2** and **4** were not detected in the medium, but **5**, which is released to the medium independently of DbaD. **1** was only detected in very low amounts (Supplementary Figure 12A). An additional intracellular extraction of SMs from mycelia of Δ*dbaD* did not reveal the presence of any of the metabolites (Supplementary Figure 13). For the synthesis of anhydrosepedonin (**1**) the non-heme Fe(II) dioxygenase AN7893 as well as the FAD-dependent monooxygenase DbaH are needed in addition to the PKS DbaI. Interestingly, the production of **1** increased when *dbaB* was deleted, indicating that DbaB converts **1** into another compound. Antibiotic C (**2**) was not detected from strains lacking AN7893, AN7894, DbaB, DbaC, DbaH, and DbaI. To produce azanidulone (**4**) only the PKS DbaI and the FAD-dependent monooxygenase DbaH are necessary. Tripyrnidone (**5**) is not detected from strains without DbaB, DbaH and DbaI. Double deletion of *dbaB* and AN7893 abolished **1** and **2**, but **3–5** were still produced (Figure 6B).

**Figure 7:**
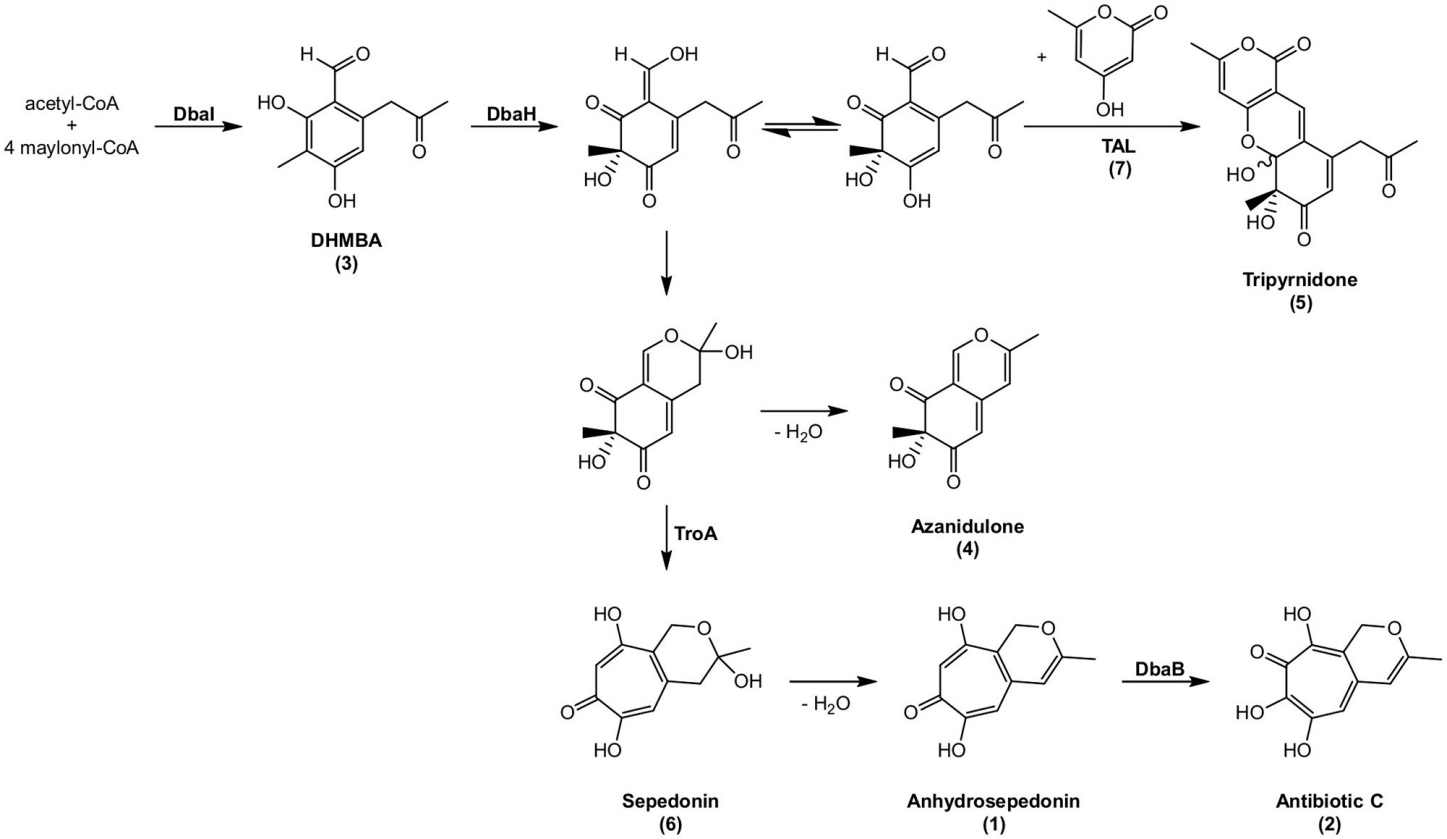
Proposed biosynthesis of compounds **1**–**6**. Compounds shown with names have been detected, compounds without names are hypothetical. The stereochemistry of **6** is not known.

Starting from DHMBA as precursor, we propose the following biosynthesis for compounds **1**, **2**, and **4** (Figure 7). DHMBA is oxidized by the FAD-dependent monooxygenase DbaH at position C-3. A ring closure follows to form the azaphilone skeleton and a subsequent spontaneous release of a water molecule leads to the formation of azanidulone (**4**). For the biosynthesis of the tropolones **1** and **2**, the carbon atom of the methyl group at position C-3 is incorporated by the non-heme Fe(II) dioxygenase AN7893 yielding sepedonin (**6**) with the 7-membered tropolone ring. A spontaneous water molecule release forms the tropolone anhydrosepedonin (**1**), which is further oxidized by the FAD-dependent monooxygenase DbaB to the tropolone antibiotic C (**2**). In summary, the PKS DbaI, DbaH and AN7893 are responsible for the formation of tropolones. Due to its key role in tropolone ring formation, AN7893 was designated TroA (tropolone-forming A).

Since the putative intermediate sepedonin (**6**) was not identified from the HPLC chromatogram at 366 nm, we searched for the presence of its mass in the LC-MS/MS data of the reference and the deletion strains. The extracted ion chromatogram at *m/z* = 225.0757 [M + H]^+^ revealed a peak with the UV/VIS spectrum of sepedonin (Supplementary Figure 2) (Supka, 1981). As expected, sepedonin (**6**) is not produced in the deletion strains of *troA, dbaA, dbaH*, and *dbaI*. In contrast, the production of sepedonin (**6**) is enhanced in Δ*dbaB* as well as in the two YCII-domain encoding deletion strains ΔAN7894 and Δ*dbaC*. The function of YCII domains was not identified yet, but they seem to have a regulatory role on SM production.

**Figure 8:**
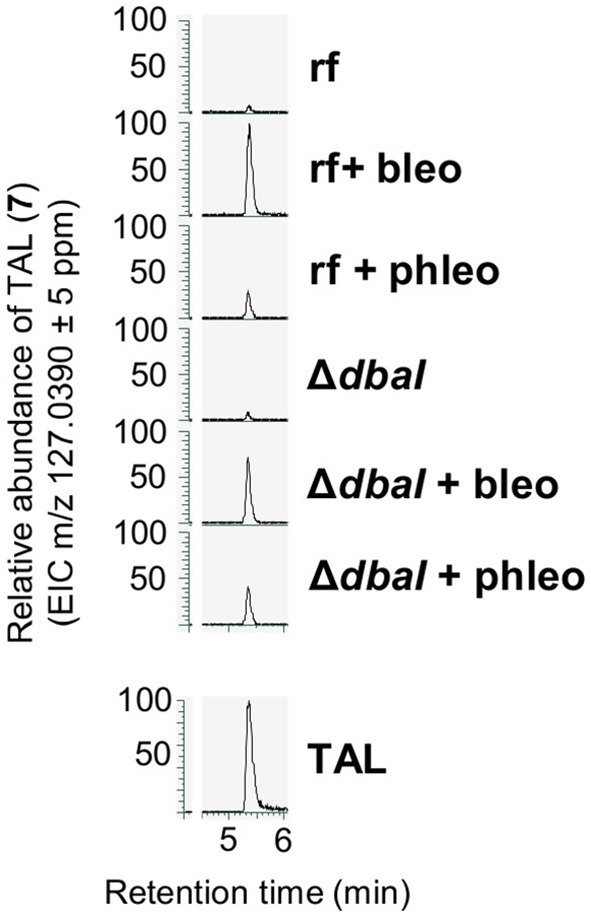
*A. nidulans* produces triacetic acid lactone (TAL, **7**). The production is increased in the presence of glycopeptide antibiotics bleomycin and phleomycin but is independent from the PKS DbaI. Reference strain AGB552 (rf) and Δ*dbaI* were cultivated in liquid medium with or without the presence of 0.1 μg/ml bleomycin or 1 μg/ml phleomycin. SMs were extracted from medium and analyzed with LC-MS/MS. Extracted ion chromatograms (EIC) for m/z = 127.0390 in positive ionization mode are shown. TAL was identified by comparison with commercial TAL (Sigma-Aldrich, lower panel).

For the biosynthesis of tripyrnidone (**5**), the oxidized DHMBA precursor presumably reacts with triacetic acid lactone (TAL). To date, TAL has not been identified from *A. nidulans*. We checked our LC-MS/MS data whether the reference strain AGB552 cultivated in bleomycin- or phleomycin-containing medium produces TAL. The extracted ion chromatogram at *m/z* = 127.0390 [M + H]^+^ revealed a peak at a retention time of 5.4 min (Figure 8). The peak was identified as TAL (**7**) by comparison with commercial TAL (Sigma-Aldrich). The TAL-producing PKS has not yet been identified in *A. nidulans*.

### Tropolones Are Iron Chelators and Can Act as Alternative Siderophores

**Figure 9:**
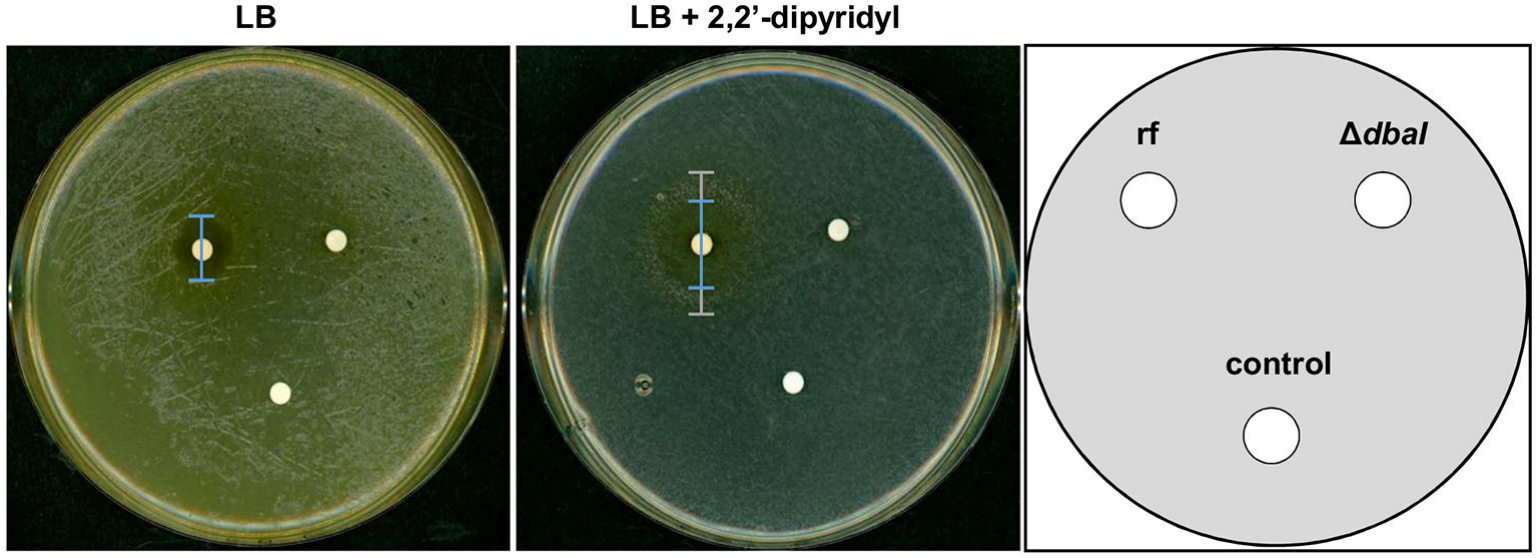
SMs of *dba*/*troA* cluster of *A. nidulans* are siderophores. Bioassay for the identification of siderophores with monoprotic keto-hydroxy bidentate ligands (KHBL). *A. nidulans* reference strain AGB552 (rf) and Δ*dbaI* were cultivated in liquid medium with phleomycin to induce the tropolone production. The metabolites were extracted, dissolved in methanol and spotted on filter disks. Pure methanol served as negative control. Solid LB medium without and with the iron-chelator 2,2'-dipyridyl (600 μM) were inoculated with *M. morganii* SBK3. The filter disks containing the metabolite extracts were placed on the agar plates, which were cultivated for 3 days at 37°C. The inhibition zone on LB without 2,2'-dipyridyl was 1.6 ± 0.1 cm (*p* ≤ 0.01, *n* = 4) for the reference strain AGB552 (rf) in comparison to 0.2 ± 0.4 cm for Δ*dbaI*. On LB with 2,2'-dipyridyl the rf extract induces an inhibition zone of 2.6 ± 0.4 cm (***p ≤ 0.001, *n* = 4) (blue bar) surrounded by a growth zone of 3.9 ± 0.5 cm (*p* ≤ 0.001, *n* = 4) (gray bar). No inhibition neither a growth zone was detected around the extract from Δ*dbaI* in presence of 2,2'-dipyridyl [*n* = 4, for significance testing a *T*-test (ri-rj) was used].

Theoretically, three tropolone molecules can form a complex with one Fe^3+^ ion. The iron binding activity of anhydrosepedonin (**1)** was shown earlier (Supka, 1981). To test whether the *dba* cluster produced tropolones can function as alternative siderophores, a bioassay for detection of siderophores with keto-hydroxy bidentate ligands was used (Thieken and Winkelmann, 1993). *M. morganii* SBK3 is a bacterium which uses siderophores with keto-hydroxy bidentate ligands for growth. Filter discs with *A. nidulans* SM extracts from reference strain AGB552 and Δ*dbaI* were put on LB agar plates supplemented with the iron chelator 2,2'-dipyridyl and inoculated with *M. morganii*. LB plates without 2,2'-dipyridyl were used as control. If the extract contains siderophores with keto-hydroxy bidentate ligands, a Morganella growth zone can be detected. The bioassay shows that *M. morganii* grows on the whole LB plate without 2,2'-dipyridyl but an inhibition zone of 1.6 ± 0.1 cm (*p* ≤ 0.01, *n* = 4) is visible for the extract of the reference strain, which is not present for the Δ*dbaI* extract (Figure 9). On plates where all free iron is bound by 2,2'-dipyridyl, *M. morganii* is able to grow in 3.9 ± 0.5 cm (*p* ≤ 0.001, *n* = 4) distance around the reference strain extract, whereas no growth is detected around the Δ*dbaI* extract. However, an inhibition zone is detected 2.6 ± 0.4 cm (*p* ≤ 0.001, *n* = 4) around the reference strain extract, surrounded by the growth zone. No bacterial growth can be observed close to the extract from the PKS deletion strain Δ*dbaI* neither to the methanol control. These results show that the tropolones produced by the *A. nidulans* AGB552 upon induction of the *dba*/*troA* gene cluster by phleomycin serve as siderophores and provide the bacteria with iron at low SM concentrations. At high concentrations, the SMs produced by the *dba*/*troA* cluster have an inhibitory effect on growth of the Gram-negative *M. morganii*.

### Anhydrosepedonin and Antibiotic C Inhibit Growth of Streptomyces spp.

**Figure 10:**
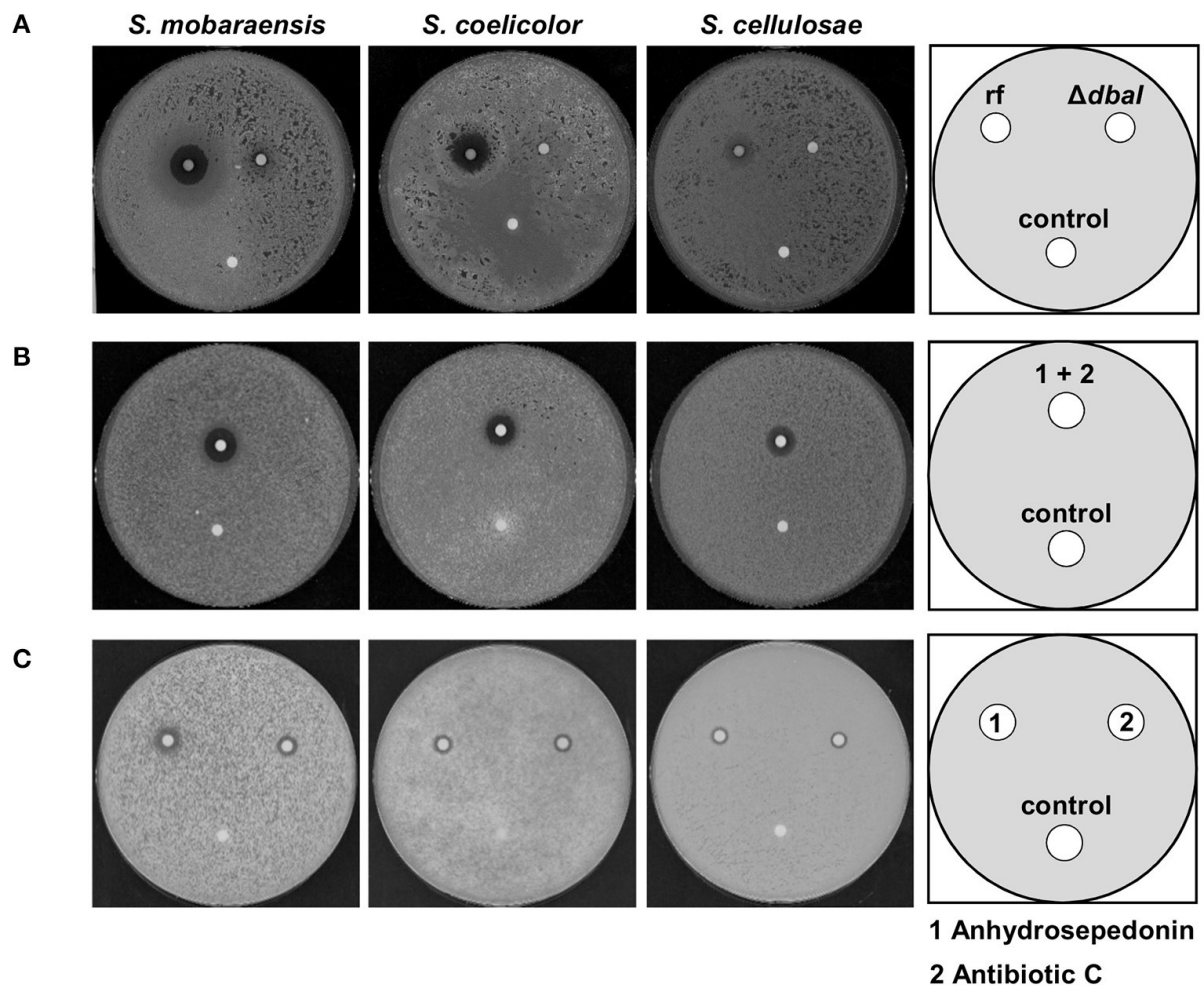
Anhydrosepedonin/antibiotic C inhibit the growth of *Streptomyces* spp. Bioactivity tests with 2 × 10^6^ spores of *S. mobaraensis, S. coelicolor*, and *S. cellulosae* distributed on whole GYM medium plates. **(A)** The SM extract of *A. nidulans* reference strain AGB552 (rf) and Δ*dbaI* was dissolved in methanol and 10 μl were spotted in a filter disk which was incubated with the inoculated plates for 2 days at 30°C. The extract of the reference strain induces a growth inhibition zone for all tested Streptomyces strains. Methanol served as control. **(B)** Anhydrosepedonin/antibiotic C were isolated from fungal extracts and 12.5 μl of the extract (stock concentration 2 mg/ml) resuspended in methanol was spotted on a filter disk and methanol served as control. The inhibition zone induced by anhydrosepedonin/antibiotic C is 1.9 cm (*S. mobaraensis*), 1.7 cm (*S. coelicolor*) and 1.5 cm (*S. cellulosae*) after 2 days of incubation at 30°C. **(C)** Anhydrosepedonin (**1**) and antibiotic C (**2**) were purified and tested separately. The compounds were dissolved in methanol to a final concentration of 2 mg/ml. Filter disc were coated with 12.5 μl extract and incubated with the Streptomyces inoculated GYM plates for 2 days at 30°C. Both metabolites induce an inhibition zone with a diameter of 1.4 cm (*S. mobaraensis*), 1 cm (*S. coelicolor* and *S. cellulosae*). Methanol served as control.

Confrontation experiments of *A. nidulans* and Streptomyces species on solid medium showed an inhibition zone (Figure 1). SM extraction revealed that *A. nidulans* produces the tropolones anhydrosepedonin and antibiotic C from the *dba*/*troA* cluster in response to either co-cultivation with *S. mobaraensis* or by media supplementation with the glycopeptides bleomycin and phleomycin. Hence, it was tested whether the SM extract from an *A. nidulans* culture supplemented with phleomycin would inhibit growth of Streptomyces species. Filter disks containing the extract of the reference strain AGB552 and the PKS deletion strain Δ*dbaI* were incubated with spores of different Streptomyces strains. The tested Streptomyces strains show a zone of inhibition around the filter disk containing the SM extract of the Aspergillus reference strain, whereas no inhibition zone was detected around the control filter disk containing methanol (Figure 10A). For the extract of the PKS deletion strain, only a minor or no inhibition zone was detected. These results revealed that the SM extract of the reference strain has a *dba*-cluster dependent antibacterial activity on Streptomyces strains.

To test whether the antibacterial activity is based on anhydrosepedonin (**1**) and/or antibiotic C (**2**), which are components of the reference strain extract, these metabolites were isolated from *A. nidulans* (Supplementary Figure 14). A filter disk was coated with either the pure compounds or a mixture of both (Figures 10B,C). Both purified compounds as well as the mixture inhibited bacterial growth.

Furthermore, anhydrosepedonin (**1**) and antibiotic C (**2**) were subjected to a broad range bioactivity test pipeline. The bacteria *B. subtilis* (DSM10), *S. aureus* (DSM346), *A. baumanii* (DSM30008), *C. violaceum* (DSM30191), *E. coli* (DSM1116) and *P. aeruginosa* (PA14), the mycobacterium *M. smegmatis* (ATCC700084) and the fungi *C. albicans* (DSM1665), *S. pombe* (DSM70572*), M. hiemalis* (DSM2656), *P. anomala* (DSM6766) and *R. glutinis* (DSM10134) were used. Additionally, the mouse fibroblast cell line L929, human cervical cancer cell line KB 3.1, human epidermoid carcinoma cell line A431, human lung carcinoma cell line A549, human prostate carcinoma cell line PC-3, human ovarian adenocarcinoma cell line SKOV-3 and human breast adenocarcinoma cell line MCF-7 were used. Antibiotic C (**2**) showed a broad, weak activity against all the tested organisms, except for *P. aeruginosa*, and showed strong cytotoxic activity against the tested cell lines with the highest activity against the cell line from human breast adenocarcinoma with an IC_50_ of 0.041 μg ml^−1^. Comparison showed lower activities for the tested anhydrosepedonin (**1**) (Table 2).

**Table 2:** Minimum inhibitory concentration (MIC) assay and cytotoxicity assay of **1** and **2**.

**Organism**	**Strain number**	**MIC (μg ml** ^**−1**^ **)**		**Positive control (μg ml^**−1**^)**
		**Anhydrosepedonin (1)**	**Antibiotic C (2)**	
*B. subtilis*	DSM10	66.6	33.3	8.3 O
*S. aureus*	DSM 346	33.3	16.6	0.21 O
*A. baumanii*	DSM 30008	n.i.	16.6	0.26 C
*C. violaceum*	DSM 30191	66.6	8.3	0.42 O
*E. coli*	DSM 1116	n.i.	33.3	3.3 O
*P. aeruginosa*	PA 14	n.i.	n.i.	0.42 G
*M. smegmatis*	ATCC 700084	66.6	33.3	1.7 K
*C. albicans*	DSM 1665	n.i.	33.3	8.3 N
*S. pombe*	DSM 70572	n.i.	33.3	4.2 N
*M. hiemalis*	DSM 2656	66.6	33.3	4.2 N
*P. anomala*	DSM 6766	n.i.	66.6	8.3 N
*R. glutinis*	DSM 10134	n.i.	33.3	2.1 N
**Cell line**	**Strain number**	**IC**_**50**_ **(μg ml**^**−1**^**)**	**Positive control (μg ml** ^**−1**^ **)**
		**Anhydrosepedonin (1)**	**Antibiotic C (2)**	**Epothilone B**
Mouse fibroblasts	L929 (ACC2)	0.81	0.085	0.00084
Human endocervical adenocarcinoma	KB3.1 (ACC158)	0.95	0.089	0.000027
Human epidermoid carcinoma	A431 (ACC91)	1.6	0.26	0.000026
Human lung carcinoma	A549 (ACC107)	0.52	0.08	0.000034
Human prostate carcinoma	PC-3 (ACC465)	2.4	0.3	0.000048
Human ovarian adenocarcinoma	SKOV-3	2.0	0.084	0.00013
Human breast adenocarcinoma	MCF-7 (ACC115)	0.73	0.041	0.000015

*n.i., no inhibition; O, oxytetracycline; C, ciprobay; G, gentamicin; K, kanamycin; N, nystatin*.

Taken together, the soil fungus *A. nidulans* produces the SMs anhydrosepedonin (**1**) and antibiotic C (**2**) from the *dba*/*troA* gene cluster, which have an antibiotic activity against Streptomycetes and a range of different other microorganisms.

## Discussion

Secondary metabolites are used for defense, communication and competition of soil communities including microorganisms as well as their predators (Linares et al., 2006; Granato et al., 2019; Gerke et al., 2020; Liu et al., 2021). Fungi and bacteria are in a continuous rivalry in consumption of substrates in soil (Boer et al., 2005). This leads to a selection pressure on bacteria to compete for easily degradable nutrients. The result is a broad repertoire of antifungal strategies like antibiotic or nutrient sequestering factors such as iron-chelating siderophores (Boer et al., 2005), which were both in focus of this study. The enormous arsenal of secondary metabolites produced by fungi and bacteria contributes to their ecological success in colonizing almost all habitats (Bills and Gloer, 2016).

We uncovered an inter-species competition between the fungus *A. nidulans* and the bacterium *S. mobaraensis*. The latter produces the antifungal glycopeptide bleomycin, which was identified as chemical signal for the activation of the tropolone-producing gene cluster *dba*/*troA* in *A. nidulans*. Bleomycin and the related phleomycin show antifungal activity against Aspergilli (Austin et al., 1990; Moore et al., 2003). Both glycopeptides cause DNA double strand breaks at unmethylated sites and single strand breaks at inverted repeat sequences in a yet unknown mechanism (Kross et al., 1982; Hertzberg et al., 1985; Moore, 1989; Ueda et al., 1992). The mechanism of the bacterium-derived glycopeptide-mediated activation of the fungal *dba*/*troA* cluster and therefore tropolone synthesis are yet unknown. Several fungal SM gene clusters like *ors* (orsellenic acid)*, dba, cic* (cichorine), *mic* (microperfuranone) and *eas* (emericellamide), which are controlled by the transcription factor BasR, were induced by bacterial signals upon co-cultivation of *A. nidulans* with *S. rapamycinicus* (Fischer et al., 2018). It will be interesting to examine whether bleomycin or phleomycin induce the expression for tropolone synthesis in a *basR* dependent manner. *Vice versa*, it will be interesting to analyze, whether fungal tropolones are capable of inducing the glycopeptide production of *S. mobaraensis*.

Whereas, in *A. nidulans* cultures supplemented with bleomycin only anhydrosepedonin and antibiotic C were detected, the supplementation with phleomycin additionally led to the identification of azanidulone (**4**), tripyrnidone (**5**), sepedonin (**6**) and TAL (**7**). Compound **4** was isolated before from the ascomycete *D. concentrica* (Buchanan et al., 1995) and the ascomycete *Nigrospora* sp. YE3033 (Zhang et al., 2016). An *in vitro* study showed that DHMBA (**3**) can be converted to azanidulone (**4**) by the addition of the enzyme AzaH of *A. niger*, which corresponds in *A. nidulans* to AN7902 coding for DbaH with 40% protein sequence identity (62% positives, 93% query coverage) (Altschul et al., 1990; Pyser et al., 2019). This suggests that no second enzyme is necessary to form the bicyclic ring of azanidulone (**4**) in *A. nidulans*. The novel scaffold of **5** was determined by NMR and high-resolution MS data and the compound was named tripyrnidone. Most likely, tripyrnidone is a PKS product of an oxidized DHMBA precursor that reacts with TAL. This study is the first to report about TAL (**7**) in *A. nidulans*. However, the non-reducing PKS predictively catalyzing this step is not yet identified. It has been shown that orsellinic aldehyde, which is structurally similar to DHMBA, can react with TAL to form precursors of melanin in *Ustilago maydis* (Reyes-Fernández et al., 2021).

This is the first report of the biosynthetic pathway of the tropolones anhydrosepedonin (**1**) and antibiotic C (**2**) in *A. nidulans*. This was elucidated by metabolite analysis of *dba*/*troA* single deletion strains (Figures 6A, 7). These mutants did not show alteration of their phenotype on medium without glycopeptides supporting that the *dba* cluster is silent under laboratory conditions (Gerke et al., 2012). We showed that the non-heme Fe(II) dioxygenase AN7893 is a key enzyme in the production of the tropolones anhydrosepedonin and antibiotic C in *A. nidulans* and therefore denoted AN7893 as TroA. Deletion of *troA* abolished the production of both compounds but not the production of the other *dba*-dependent metabolites **3–5** (Figure 6). It was recently discovered that non-heme Fe(II) dioxygenases are responsible for the incorporation of a C-atom to form the typical seven-membered ring of tropolones (Davison et al., 2012; Cox and Al-Fahad, 2013). The ascomycete *Talaromyces stipitatus* produces the tropolone stipitatic acid. The biosynthetic enzymes were identified as the non-reducing PKS TropA, the FAD-dependent monooxygenase TropB, the non-heme Fe(II) dioxygenase TropC as well as the cytochrome P450 monooxygenase TropD (Davison et al., 2012). A protein homology search revealed that the *dba/troA* cluster is fully conserved in *T. stipitatus* with protein sequence identities of 58–92% (Table 3; Supplementary Figure 15). By querying the JGI fungal genome database MycoCosm (Grigoriev et al., 2014) it was found that about 7.5% of Aspergillus species contain the *dba/troA* gene cluster. However, outside the *Aspergillus*, the *dba/troA* gene cluster was only detected in *T. stipitatus* ATCC 10500, in *Coccidioides immitis* and *C. posadasii* (Supplementary Table 9) (Sharpton et al., 2009; Davison et al., 2012). The *dba* cluster of *Coccidioides* spp. is rearranged and lacks an AN7901 (*dbaG*) orthologous gene (Supplementary Figure 15).

**Table 3:** Biosynthetic proteins encoded in *dba*/*troA* cluster of *A. nidulans* responsible for production of **1–5**.

**Name**	**Locus tag**	**Function**	**Homologous protein of *T. stipitatus* tropolone cluster**	**Protein percent (%) identity**
TroA	AN7893	Non-heme Fe(II) dioxygenase	TropC	58.33
DbaB	AN7897	FAD-dependent monooxygenase	TropD	87.24
DbaH	AN7902	FAD-dependent monooxygenase	TropB	59.82
DbaI	AN7903	Non-reducing PKS	TropA	81.88

*Protein percent identities were determined by utilizing the clustal omega multiple protein sequence alignment tool (EMBL-EBI) and sequences were obtained from FungiDB (Sievers et al., 2011; Basenko et al., 2018)*.

Besides biosynthetic genes, the *dba* cluster encodes genes for transcription control and transport. Deletion of the putative MFS transporter gene *dbaD* abolished the extracellular detection of **2** and **4**, but these compounds were also not detected in intracellular extracts (Supplementary Figure 13). This indicates that instead of being a plasma membrane transporter, DbaD might be an organelle membrane transporter. For several fungal SMs it was shown that biosynthetic enzymes are co-compartmentalized and the biosynthetic intermediates are shuttled between different organelles in order to channel precursors and to promote pathway efficiency (Kistler and Broz, 2015).

Tropolones are rare natural products comprising a seven-membered aromatic ring, also called the tropolonoid motif, and are found in fungi, bacteria, and plants (Bentley, 2008; Davison et al., 2012; Cox and Al-Fahad, 2013; Guo et al., 2019). They show a wide range of bioactivity including antiviral, antitumor, bactericidal and bacteriostatic properties against a wide range of bacteria (Trust, 1975; Nagao et al., 2006; Ononye et al., 2013; Tavis and Lomonosova, 2015). Inhibited proliferation of some (pathogenic) fungi and an insecticidal effect are described against *Tyrophagus putrescentiae, Dermatophagoides farina*, and *Captotermes formosanus* (Morita et al., 2003, 2004; Quang et al., 2010; Azadbakht et al., 2020). In this study, the produced tropolones anhydrosepedonin (**1**) and antibiotic C (**2**) show antibacterial activity against *S. mobaraensis*. Further MIC tests revealed bioactivity against a broad range of bacteria and fungi as well as cytotoxicity against all tested cell lines (Table 2). The discovery of the biosynthetic pathways and novel compounds that are bioactive against different microorganisms is an interesting starting point for future research.

Streptomyces are rarely pathogenic to humans with only a few pathogenic species, wherefore the tropolone antibiotic effect against Streptomyces is rather irrelevant for pharmaceutical use. The reported broad spectrum of bioactivity of tropolones (Trust, 1975; Morita et al., 2003; Azadbakht et al., 2020; Duan et al., 2020) and our MIC tests suggest an promising medical potential of these identified compounds against other microorganisms and cancer cell lines. Biotechnological applications of **1** and **2** might be facilitated by our discovery of the biosynthetic pathway and the physiological condition under which the *dba/troA* cluster is active. The production of these tropolones under laboratory conditions using phleomycin is an advantage for further studies on their bioactive potential.

Tropolones not only show anti-microbial, anti-insecticidal and anti-cancer potential but have the ability of metalloprotease inhibition. Therefore, the bioactivity of different tropolones is often referred to their metal-chelating and redox potential (Morita et al., 2003; Chen et al., 2019). Our experiments show that the extracted tropolones anhydrosepedonin (**1**) and antibiotic C (**2**) can inhibit growth of *S. mobaraensis, S. coelicolor*, and *S. cellulosae*. Three tropolone molecules can bind one Fe^3+^ and therefore serve as siderophores (Supka, 1981). Siderophores are chelators produced by microorganisms for the uptake and storage of iron (Kraemer, 2004). Iron is an essential trace element for many vital pathways and cellular functions where it serves as cofactor for numerous enzymes (Coffey and Ganz, 2017; Misslinger et al., 2021). According to their structure, tropolones belong to the rare group of keto-hydroxy bidentate ligand (KHBL) siderophores. A bioassay for the detection of KHBL siderophores (Thieken and Winkelmann, 1993) showed that *dba*-dependent metabolites such as the tropolones anhydrosepedonin (**1**) and antibiotic C (**2**) are KHBL siderophores at least for *M. morganii* (Figure 9). Whether *A. nidulans* utilizes the tropolones as alternative siderophores is still speculative. Previously, it was shown that the biosynthetic precursor sepedonin (**6**) can function as KHBL siderophore as well (Thieken and Winkelmann, 1993). Interestingly, the KHBL siderophore bioassay further revealed that the *dba*/*troA* SMs have an antibiotic effect on the Gram-negative *M. morganii*. Around the SM-coated filter disc a SM concentration gradient exists due to diffusion. In short distance to the filter disc high metabolite concentrations are present inhibiting the growth of *M. morganii*, whereas in long distance to the filter disc with low SM concentrations a growth zone is present, showing that the *dba*/*troA* SMs can provide *M. morganii* with iron (Figure 9). A myriad of applications are described for siderophores (Ahmed and Holmström, 2014; Albelda-Berenguer et al., 2019). Siderophores are used in bioremediation of metal pollution and growth enhancement of plants. Siderophores have medical applications in reducing the iron overload during blood transfusion, or as sideromycins in which a siderophore is covalently bound to an antibiotic (Braun et al., 2009). In *A. nidulans* the siderophore ferricrocin is known for its iron storage properties and is involved in oxidative stress control (Eisendle et al., 2006; Albelda-Berenguer et al., 2019). It is interesting to further investigate the advantage of the newly identified siderophores of *A. nidulans*.

Even in well-studied organisms, the identification of the complete arsenal of SMs proves difficult, because the expression of the responsible biosynthetic gene clusters is often silenced under laboratory conditions. SMs are specialized chemicals, which are only active under certain environmental stimuli, developmental stages or due to genetic manipulation (Calvo et al., 2002; Gerke and Braus, 2014). Activation of certain biosynthetic gene clusters that produce metabolites used for signaling and defense in nature can be achieved by co-cultivation of different species (Schroeckh et al., 2009). The co-cultivation of this study helped to understand the physiological conditions needed to activate the *dba*/*troA* gene cluster. Thereby, we found otherwise overlooked bioactive compounds with potential relevant pharmacological use. The plethora of possible microbial interactions in the complex soil ecosystem was also used in previous co-cultivation studies to identify several new compounds. For example, *A. niger* in co-cultures with *S. coelicolor* resulted in the production of ten new compounds that were not present in monocultures (Wu et al., 2015). When co-cultivated with *Streptomyces* sp., *Aspergillus flavipes* produces cytochalasans, which are cytotoxic to the bacterium and thus provide an advantage for the fungus (Yu et al., 2016). *S. rapamycinicus* activated the cryptic meroterpenoid pathway synthesizing the beforehand unknown prenylated polyketide fumicycline in *A. fumigatus* (König et al., 2013). Another study showed that the physical interaction of *A. nidulans* with *S. rapamycinicus* induced the expression of three neighbored biosynthetic gene clusters, the PKS-containing *ors* gene cluster, producing orsellinic acid and F-9775A/B, the NRPS-containing *atn* gene cluster, producing aspercryptin, as well as the PKS-containing *dba* gene cluster producing DHMBA (Schroeckh et al., 2009; Gerke et al., 2012; Chiang et al., 2016). Interestingly, AN7893 (*troA*), which is indispensable for converting the *dba* cluster SMs to tropolones, was not upregulated during co-cultivation of *A. nidulans* with *S. rapamycinicus*.

Whereas, the before mentioned study proved that a physical interaction of the two microorganisms was necessary to induce SM production (Schroeckh et al., 2009), we showed that not the physical contact but the secretion of chemicals is used as signal. The advantage for *A. nidulans* is that chemicals can be detected over distances, diffusion being the limiting factor, which allows the fungus to counteract early with the production of antibacterial substances. Furthermore, the SM can have concentration-dependent functions, where it acts as a signal and toxin (Keller, 2019). This study shows that bleomycin and phleomycin have an antifungal effect as well as a signaling function by inducing the *dba* gene cluster. Furthermore, the tropolone-containing SM extract showed a concentration-dependent growth inhibition and iron-supplying effect for *M. morganii* (Figure 9). This information is important for understanding the physiological conditions of SM synthesis and their biological effect.

## Conclusion

The discovery of new pharmaceutically or agriculturally relevant natural products is a challenging task since most of these metabolites are hidden by silent biosynthetic gene clusters. The here described co-cultivation of bleomycin-producing Streptomyces with *A. nidulans* uncovered the novel metabolite tripyrnidone (**5**) as well as the azaphilone azanidulone (**4**) and the antibacterial tropolones anhydrosepedonin (**1**) and antibiotic C (**2**). We described the biosynthesis pathway with the responsible involved enzymes and showed that the products have an iron-chelating as well as a growth inhibitory function on Streptomyces species. These findings revealed an inter-species communication or competition via chemical signals in form of the SMs bleomycin and tropolones, latter with potential for next-generation drug development.

## Data Availability Statement

The original contributions presented in the study are included in the article/Supplementary Material, further inquiries can be directed to the corresponding author/s.

## Author Contributions

JG, HB, FS, and GB conceived the study and designed the experiments. JG, AK, J-PW, VG, LS, and AH performed the experiments. JG, AK, J-PW, AH, WC, and FS analyzed the data. JG, AK, J-PW, FS, and GB wrote the manuscript. All authors contributed to the study and approved the final version of the manuscript.

## Funding

Funding was provided by the German Research Council to GB (DFG grant BR 1502/19-1). We acknowledge support by the Open Access Publication Funds of the University of Göttingen.

## Conflict of Interest

The authors declare that the research was conducted in the absence of any commercial or financial relationships that could be construed as a potential conflict of interest.

## Publisher's Note

All claims expressed in this article are solely those of the authors and do not necessarily represent those of their affiliated organizations, or those of the publisher, the editors and the reviewers. Any product that may be evaluated in this article, or claim that may be made by its manufacturer, is not guaranteed or endorsed by the publisher.
