# Supplementary material for: Biosynthesis of Antibacterial Iron-Chelating Tropolones in Aspergillus nidulans as Response to Glycopeptide-Producing Streptomycetes
Source: Front Fungal Biol. 2022 Jan 3;2:777474. doi: 10.3389/ffunb.2021.777474 (PMC10512232; doi:10.3389/ffunb.2021.777474)
Supplement: Supplementary file 1 [file Data_Sheet_1.docx]

Supplementary Material

**Supplementary Figures**

**
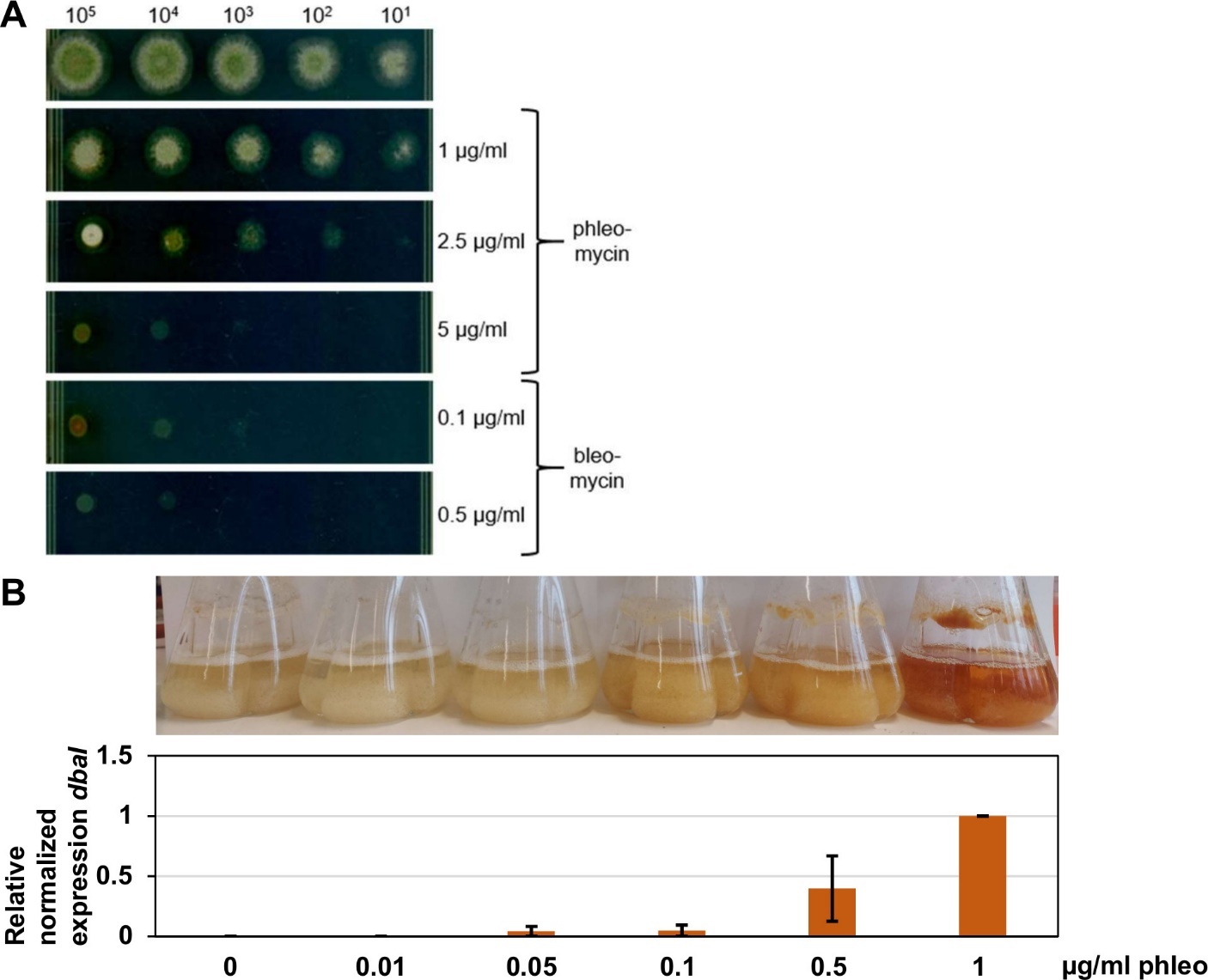
**

**Supplementary Figure 1: (A)** *A. nidulans* AGB552 was spotted in different spore concentrations (10^5^-10^1^ spores) on London Medium with different concentrations of phleomycin or bleomycin. The plates were incubated for 3 days at 30°C. **(B)** *A. nidulans* AGB552 was cultivated in liquid London Medium with different concentrations of phleomycin for two days at 30°C. The orange color gradient of the medium and mycelium is strongest at 1 µg/ml phleomycin. qPCR with extracted RNA from those cultures revealed expression of *dbaI* at 0.5 and 1 µg/ml phleomycin. Expression of the housekeeping genes *h2A, gpdA* and *rps15* were used for normalization. Error bars represent the standard error of the mean of three biological replicates.

**
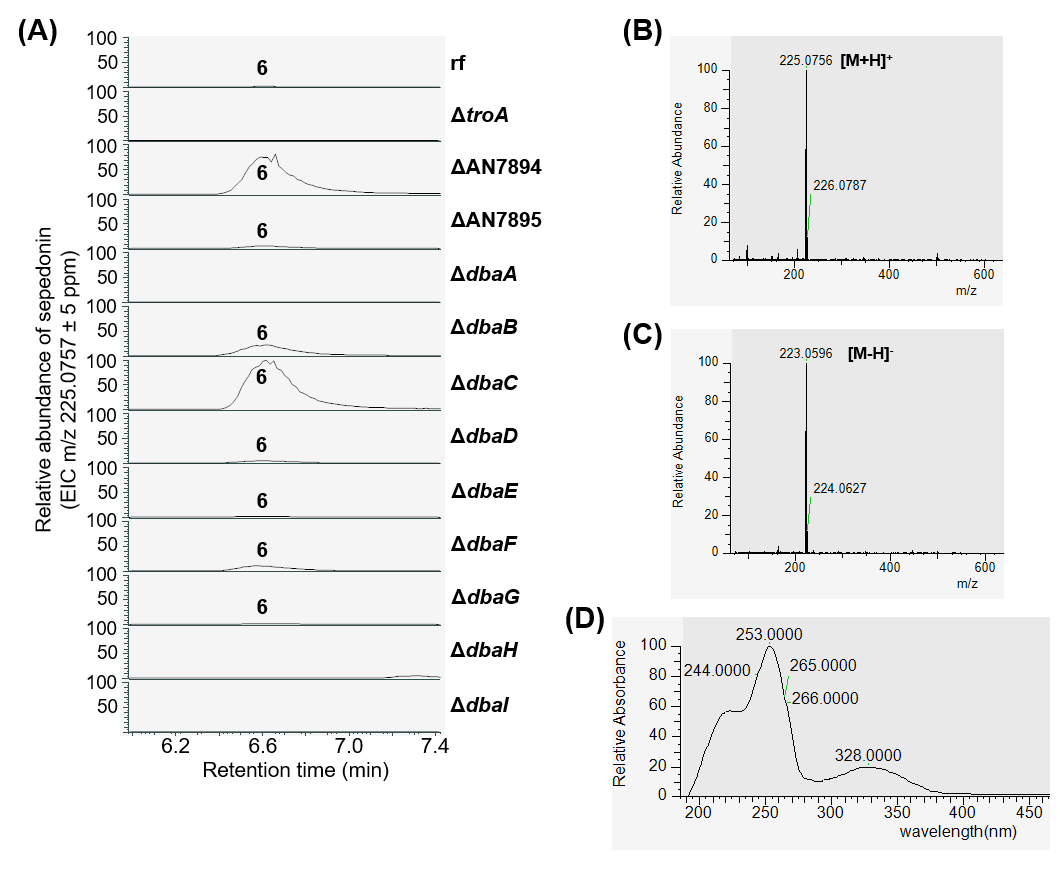
**

**Supplementary Figure 2:** The *dba*/*troA* cluster of *A. nidulans* produces the tropolone sepedonin (**6**). (**A**) The indicated strains were cultivated in liquid medium supplemented with 1 µg/ml phleomycin for 2 days. Ethyl acetate extracts of culture filtrates were analyzed with LC-MS/MS. The relative abundance of sepedonin (**6**) in the extracted ion chromatogram (EIC m/z 225.0757 ± 5 ppm) is shown. rf = reference strain AGB552. (**B**) Full MS spectrum of **6** in positive ionization mode. (**C**) Full MS spectrum of **6** in negative ionization mode. (**D**) UV/VIS spectrum of **6**.

**
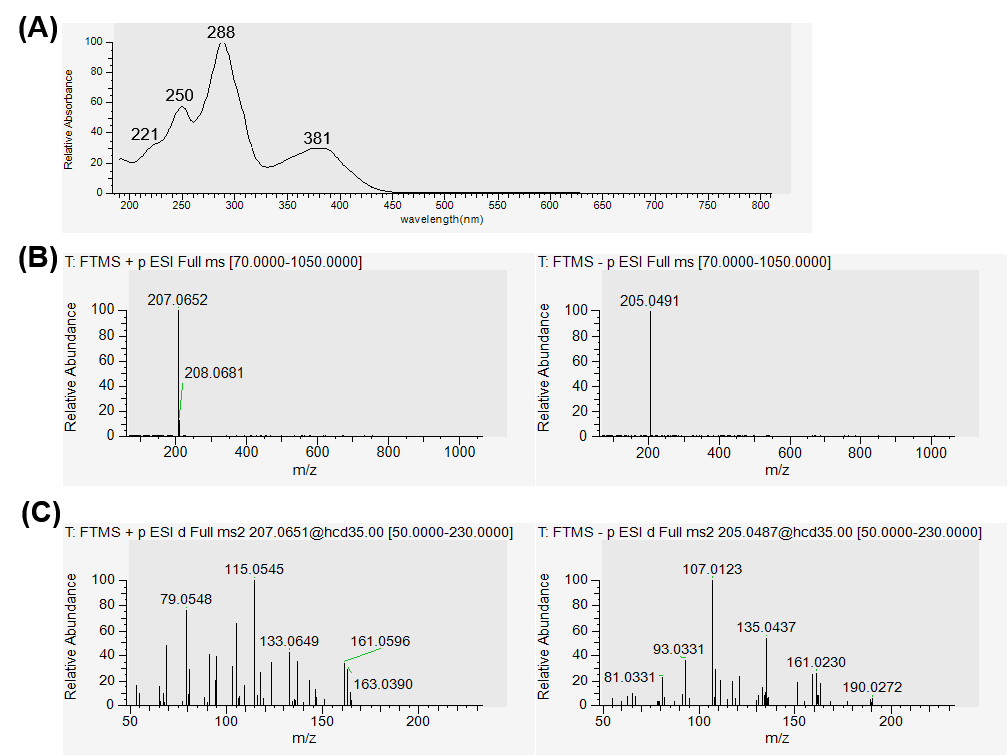
**

**Supplementary Figure 3:** UV/VIS spectrum (**A**), full MS spectra (**B**) and MS^2^ spectra (**C**) of anhydrosepedonin (**1**).

**
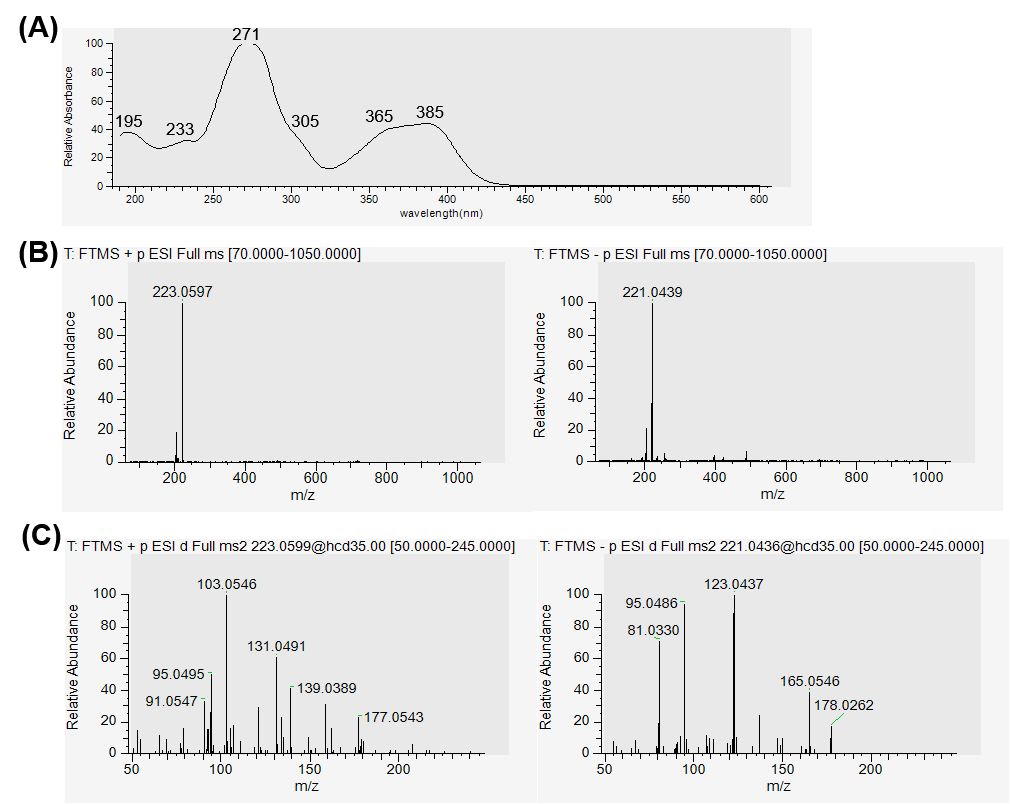
**

**Supplementary Figure 4:** UV/VIS spectrum (**A**), full MS spectra (**B**) and MS^2^ spectra (**C**) of antibiotic C (**2**).

**
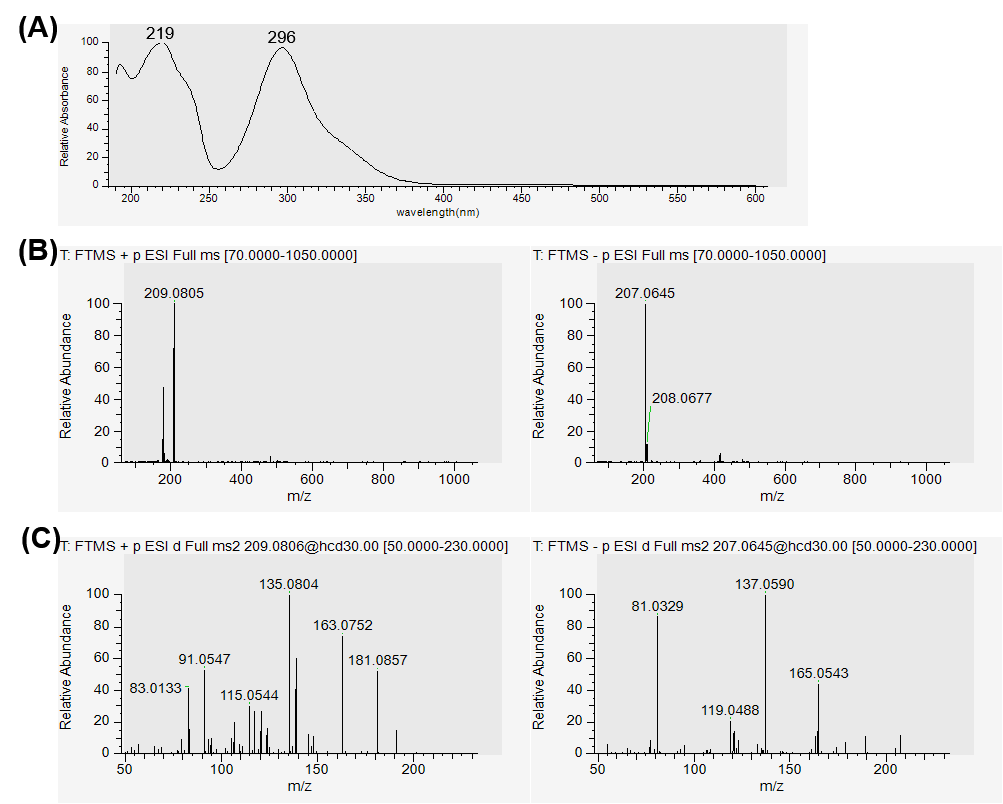
**

**Supplementary Figure 5:** UV/VIS spectrum (**A**), full MS spectra (**B**) and MS^2^ spectra (**C**) of DHMBA (**3**).

**
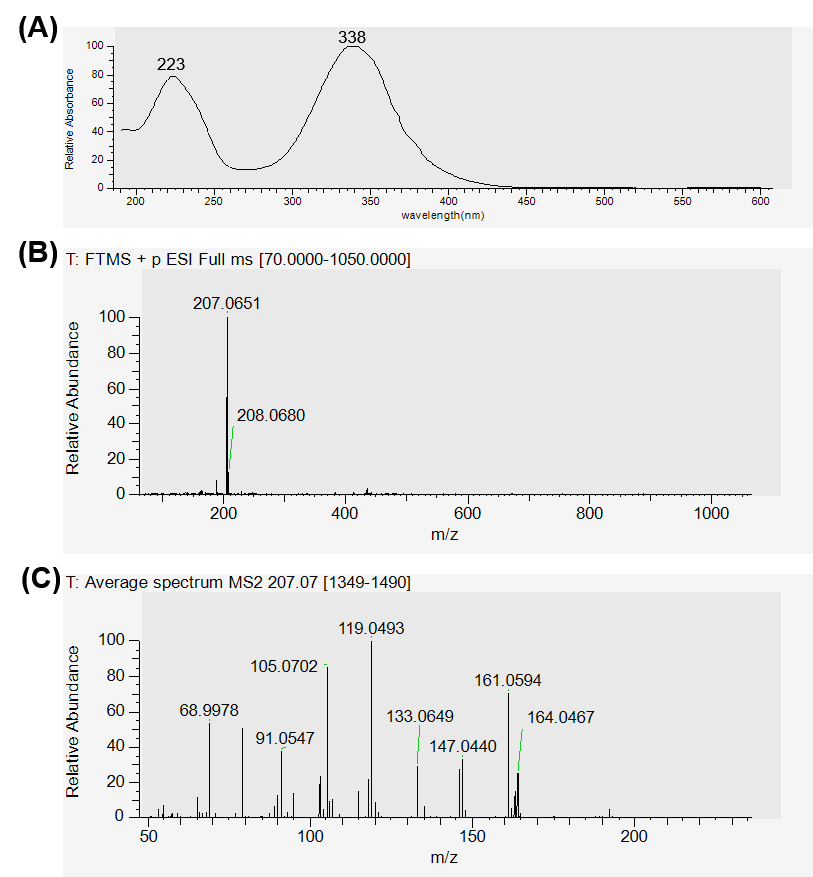
**

**Supplementary Figure 6:** UV/VIS spectrum (**A**), full MS spectrum (**B**) and MS^2^ spectrum (**C**) of azanidulone (**4**).

**Supplementary Figure 7:** CD-spectrum of azanidulone (**4**) in H_2_O


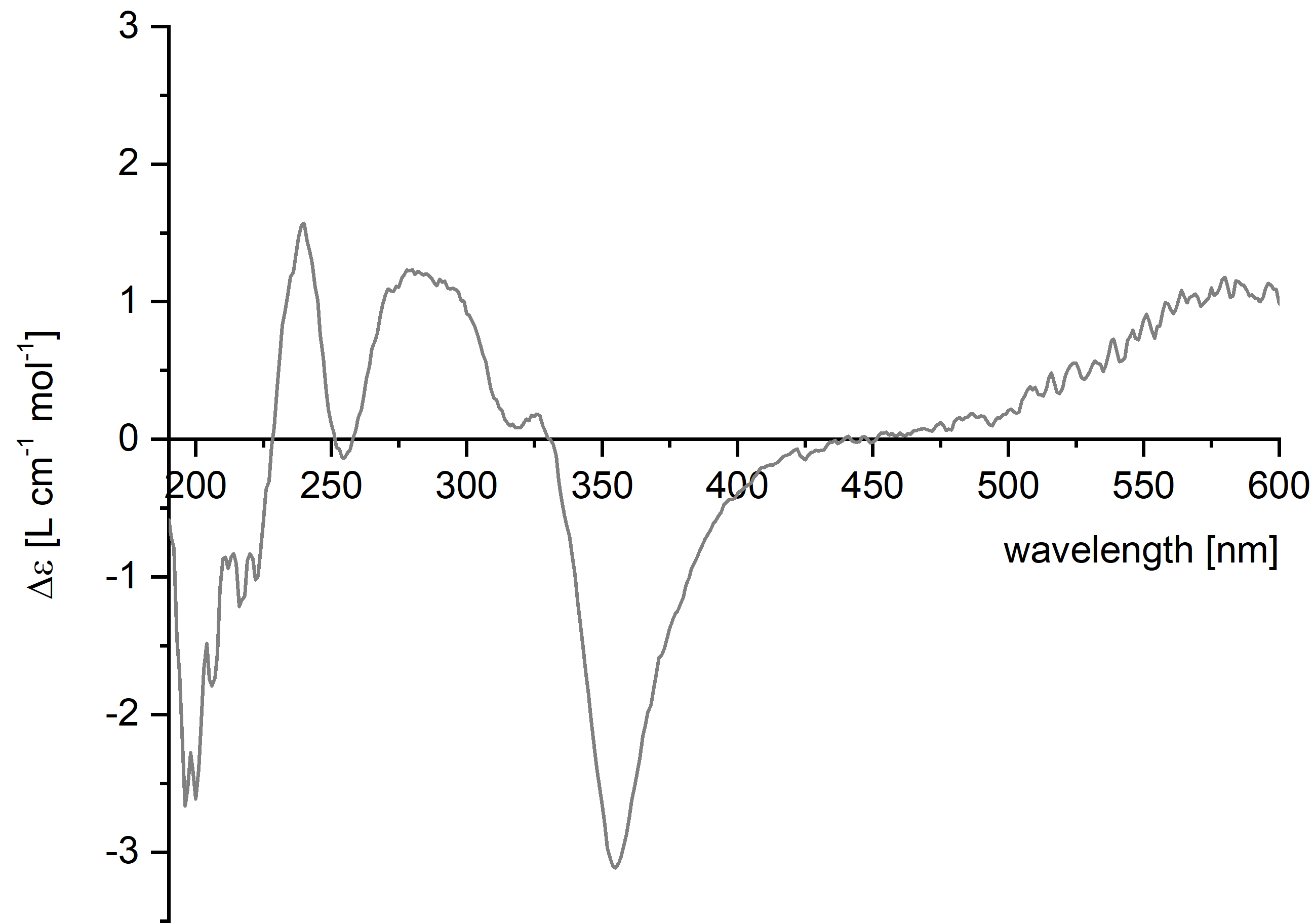


**
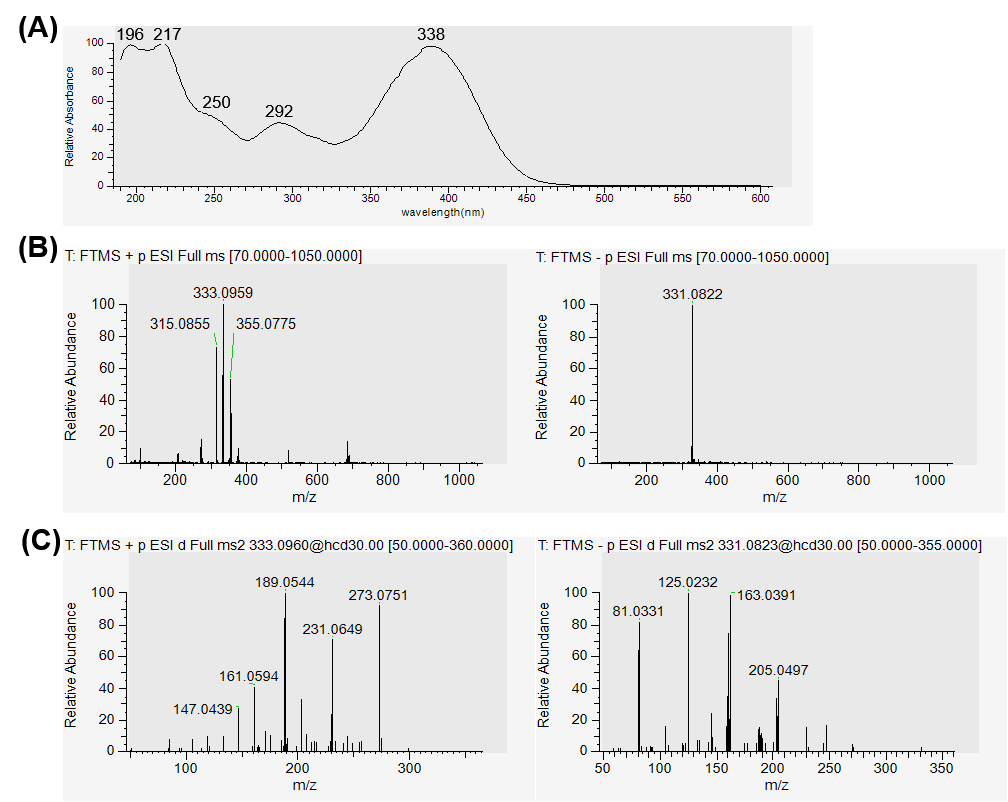
**

**Supplementary Figure 8:** UV/VIS spectrum (**A**), full MS spectra (**B**) and MS^2^ spectra (**C**) of tripyrnidone (**5**).

**
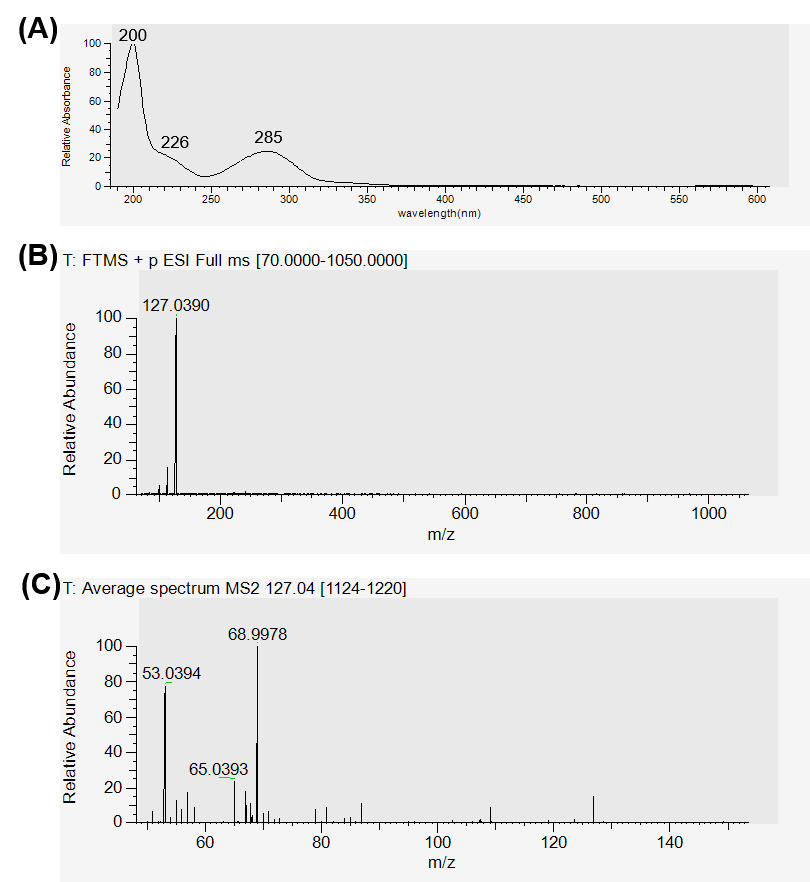
**

**Supplementary Figure 9**: UV/VIS spectrum (**A**), full MS spectrum (**B**) and MS^2^ spectrum (**C**) of triacetic acid lactone (TAL, **7**).

**Supplementary Figure 10:** Structure of anhydrosepedonin (**1**) with HMBC & NOESY correlations


**Supplementary Figure 11:** Structure of antibiotic C (**2**) with HMBC & NOESY correlations

**A**

**
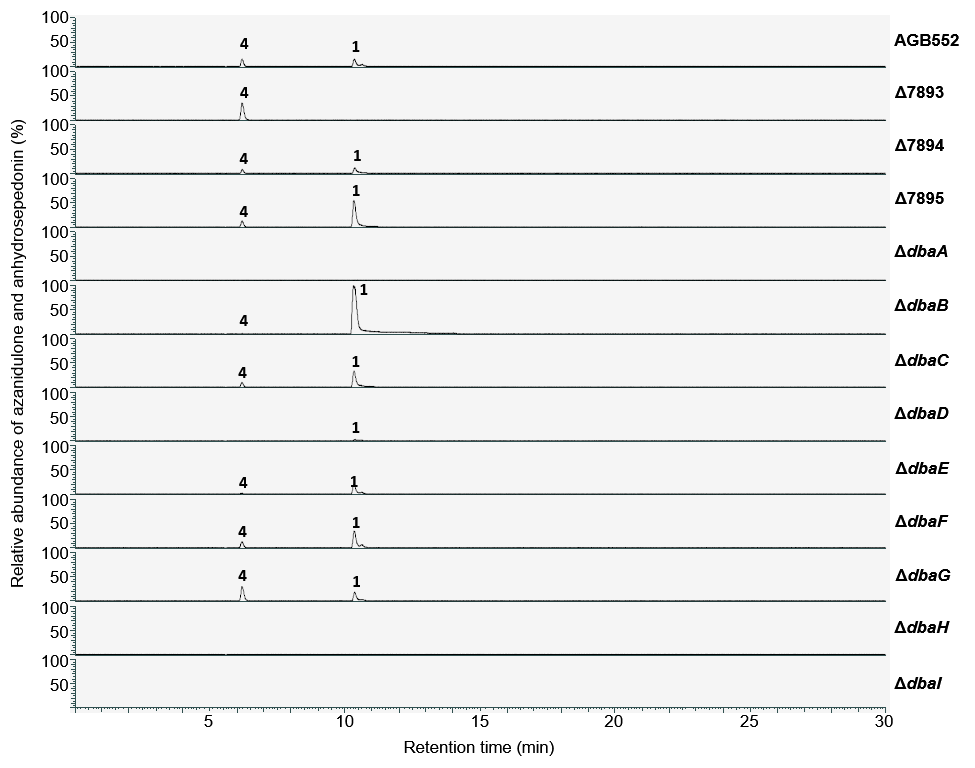
**

**B**

**
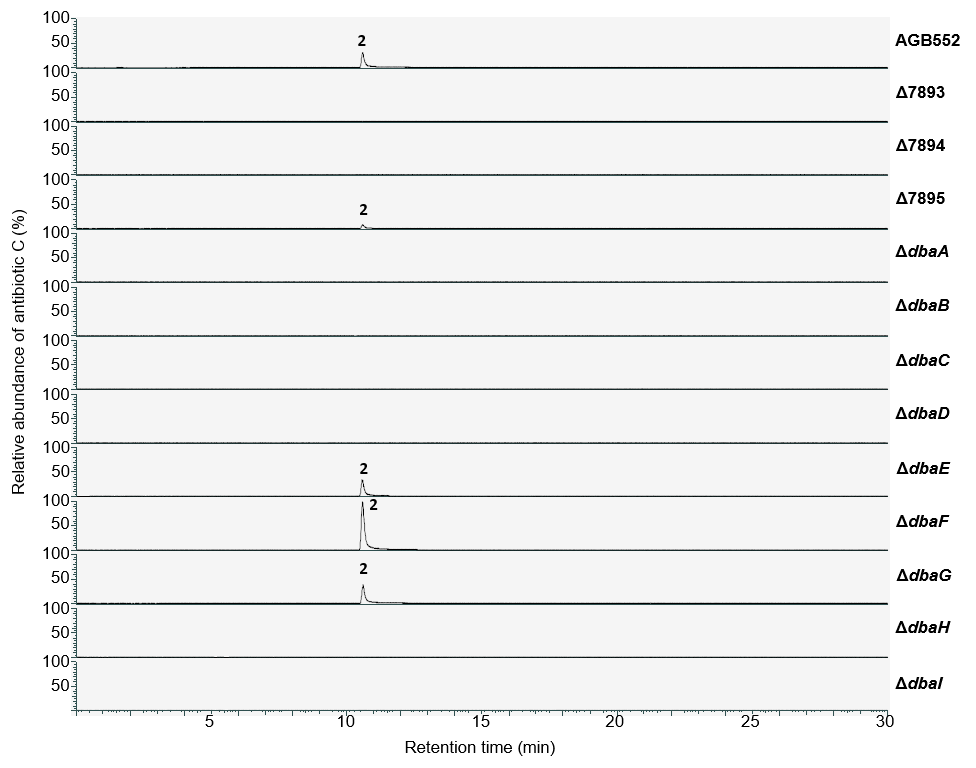
**

**C**


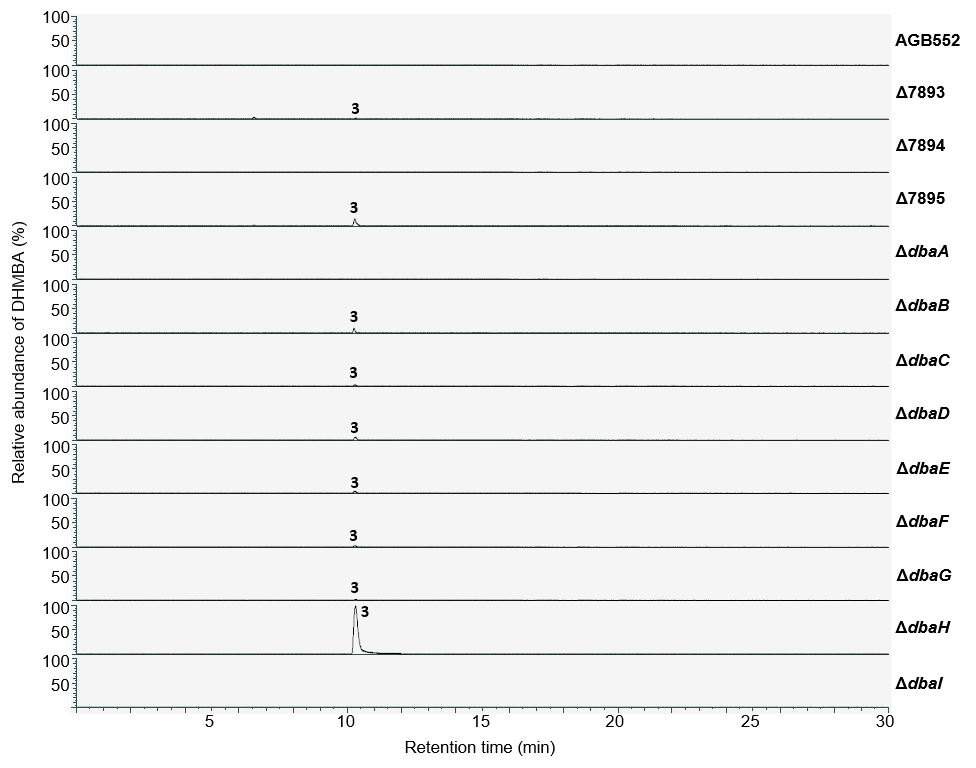


**D**


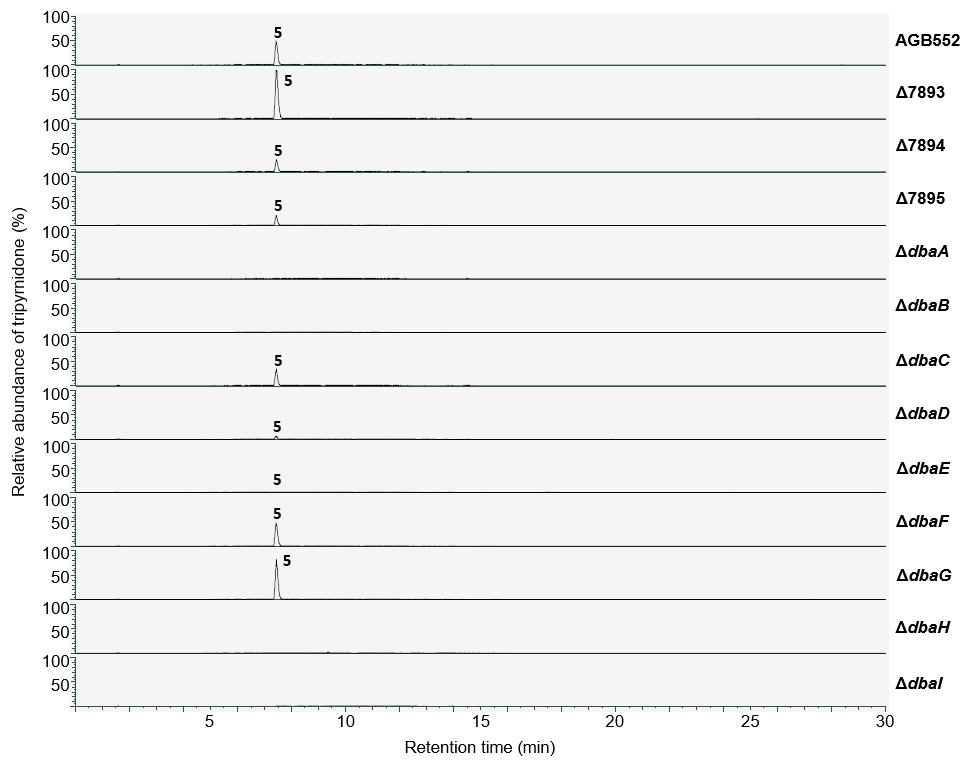


**E**


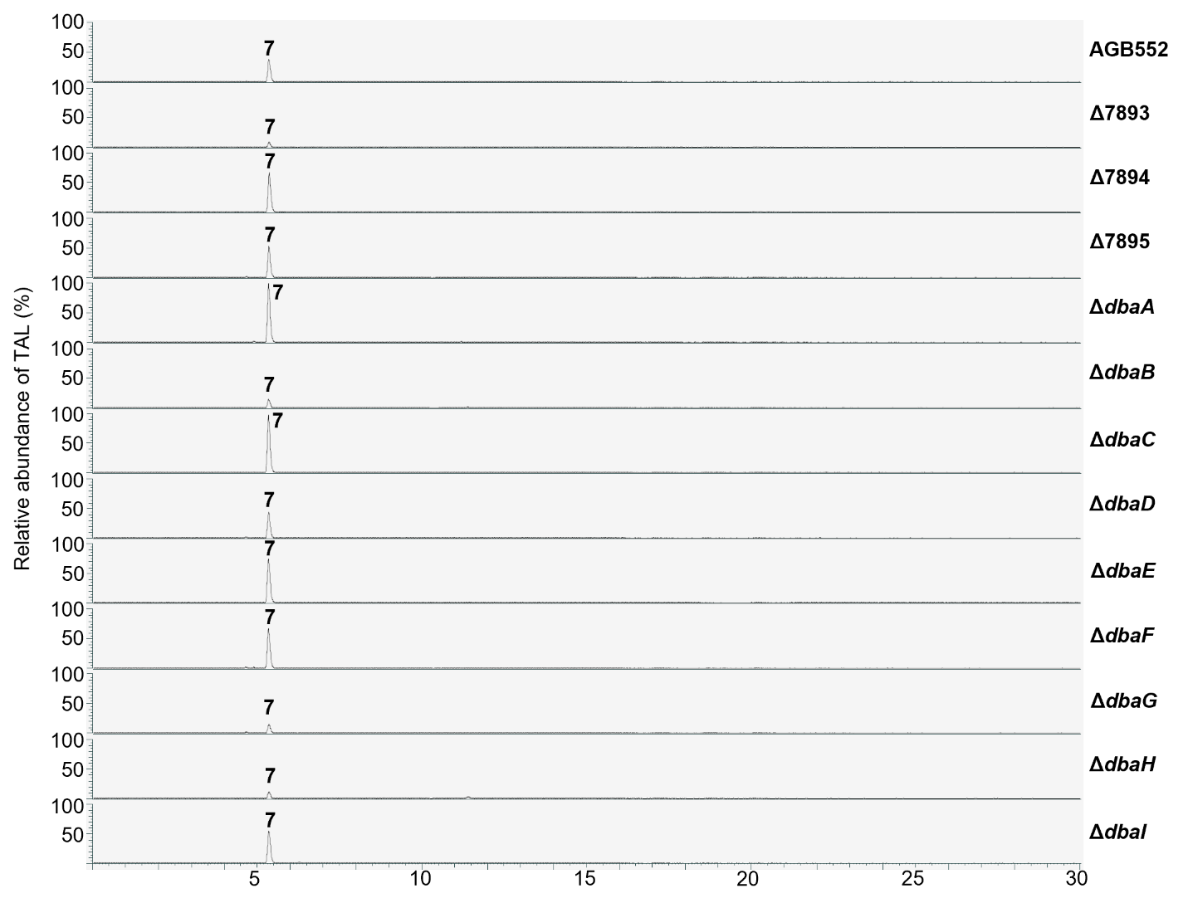


**Supplementary Figure 12:** Extracted ion chromatogram (EIC) for **(A)** azanidulone (**4**) and anhydrosepedonin (**1**) with positive ionization at m/z = 207.0652 ± 5 ppm, **(B)** antibiotic C (**2**) with positive ionization at m/z = 223.0601 ± 5 ppm, (**C**) DHMBA (**3**) with positive ionization at m/z = 209.0808 ± 5 ppm, **(D)** tripyrnidone (**5**) with positive ionization at m/z = 333.0969 ± 5 ppm and **(E)** triacetic acid lactone (TAL) (**7**) with positive ionization at m/z = 127.0390 ± 5 ppm. Indicated strains were cultivated in liquid medium supplemented with 1 µg/ml phleomycin at 30°C and 120 rpm for 2 days. Ethyl acetate extracts of culture filtrates were analyzed with LC-MS/MS.

**
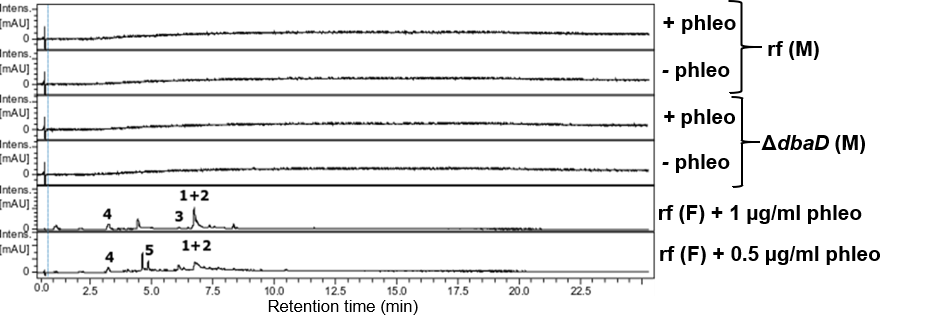
**

**Supplementary Figure 13:** The *A. nidulans* reference strain AGB552 (rf) and Δ*dbaD* were cultivated in liquid London medium supplemented with the 1 µg/ml phleomycin (if not noted otherwise) for 2 days at 30°C. Acetone extracts of mycelia (M) and ethyl acetate extracts of culture filtrates (F) were analyzed with LC-MS/MS equipped with a DAD detector. The relative absorbance at 366 nm is shown. (**1**) anhydrosepedonin, (**2**) antibiotic C, (**3**) DHMBA, (**4**) azanidulone, (**5**) tripyrnidone.


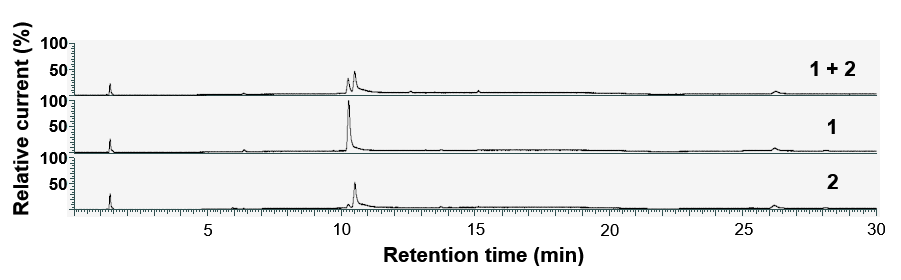
**Supplementary Figure 14:** Chromatograms of the purified metabolites anhydrosepedonin (**1**) and antibiotic C (**2**) from Figure 10B and 10C analyzed with LC-MS/MS equipped with a CAD detector. The relative current is shown.


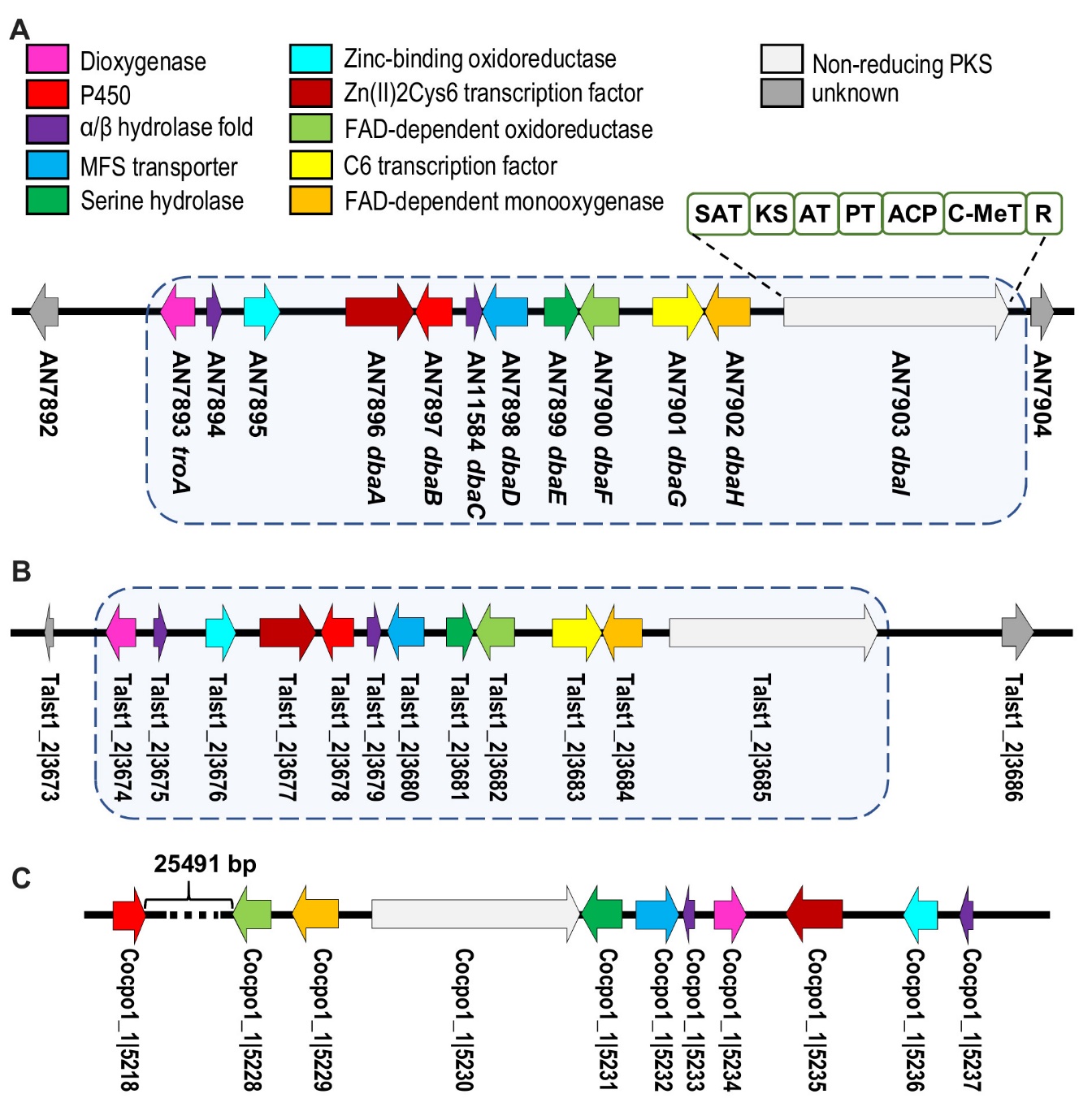
 **Supplementary Figure 15: Gene arrangement of the *dba/troA* gene cluster in *A. nidulans*, *Talaromyces stipitatus* and *Coccidioides posadasii***. The boundary of the gene cluster is boxed. The encoded protein features and function prediction are annotated with colors as indicated. **(A)** The *dba/troA* gene cluster in *A. nidulans*. SAT, starter unit acyl transferase; KS, ketosynthase; AT, acyl transferase; PT, product template; ACP, acyl carrier protein; C-MeT, C-methyl transferase; R, acyl CoA thiolester reductase. **(B)** The *dba/troA* gene cluster in *T. stipitatus* according to JGI fungal genome database MycoCosm (Davison et al., 2012; Grigoriev et al., 2014). Talst1_2|3673 is not the ortholog of AN7892, and Talst1_2|3686 is not the ortholog of AN7904. **(C)** The *dba/troA* gene cluster in *C. posadasii* according to JGI fungal genome database MycoCosm (Sharpton et al., 2009; Grigoriev et al., 2014). Cocpo1_1|5218, the ortholog of AN7897 (*dbaB*) is located ten genes (25491 base pairs (bp)) upstream of the cluster. No orthologous gene was found for AN7901 (*dbaG*).

**Supplementary Tables**

**Supplementary Table 1**: Primers used in this study.

| **Name** | **Sequence 5’🡪 3’** |
| --- | --- |
| JG680 | GACTGGATTGAGACGGAGCAAA |
| JG681 | TTCAGGACAAGGAAGACGGATG |
| JG682 | CCGAGACAGATGCGGACAGAT |
| JG683 | CAACAGGCACCCAATCCACTAA |
| JG684 | ACCCGCACACCTGGAACATAAC |
| JG685 | GAATACACATCACGCTCCCAACA |
| JG686 | GTTTCTTCGGCGGTGCTCTAAT |
| JG687 | CAGTTGGAATGGTGGGAATGAG |
| JG688 | CCAGCGGAGAAGAGGCAGATTA |
| JG689 | CATAGACGAAGCGAAAGGTGGA |
| JG690 | TCACCTACAAGGACCCCAACAC |
| JG691 | CCCGAATGACGCAAAAGAAAG |
| JG692 | GAGTCCCTCGCCGTATCAACTC |
| JG693 | CCTATGATCGCTTGTGGGGTCT |
| JG880 | ACGACGAGGGTCTTATTGTCA |
| JG881 | TAGCCTTCTCCTGGGTAGTGAA |
| JG882 | CATTGCTTTTGGTGGTGTTCT |
| JG883 | ATGCGTCCTTCTTCTTCTTCTG |
| JG884 | GCCGAGATCAAGAAGAGAAGAC |
| JG885 | GAAGCTGTTCAGAGGAGAGGTC |
| JG1437 | ACGGTATCGATAAGCTTGATGTTTAAACGCTTCTTGCACTCGACCA |
| JG1438 | TATTGACCTATAGGCCTGAGTTTCCTGCCTGTTCTAGTGCGA |
| JG1439 | TGAGCATAATATGGCCATCTTGTCCTTTACCTCAGCTTTTGTTT |
| JG1440 | CCGGGCTGCAGGAATTCGATGTTTAAACTCCTCCAATACACCTGCC |
| JG1445 | TCTCTCGCCTTACAGTGAATGA |
| JG1446 | AGTGATGGAGTGCTGAGGTTCT |
| JG1454 | AAGTTTAAACTCCGAGCGAATTTCCCGA |
| JG1455 | TTTGATTTACTGAAGTTTTGATGCT |
| JG1456 | AGGGCTCGTGTTTCTCGAGATT |
| JG1457 | GTTTAAACTTCACTCGACAGGGCCAA |
| JG1458 | GTTTAAACCAACAGTCTCCTTATTGTGA |
| JG1459 | AAATCGTAGGGATGATTTGGTAATTAAT |
| JG1460 | TGTTTGGTGTTGAAATATTATCAGA |
| JG1461 | GTTTAAACGCCTGCAAGGCCGTCG |
| JG1478 | TTGTTGACGGGACGACTGTAG |
| JG1479 | TTTGTGCGTGTAGTGAGGGTAG |
| JG1480 | AAAGTGCTGCTCAAAATCCTTG |
| JG1481 | AACTGTCTCTCGTCTCTGACTGG |
| JG1517 | AGGAATTCGATATTTGTTTAAACAGGAGGCGGCGTTGAGA |
| JG1518 | ATAGGCCTGAGATTTTTCACTGGCGGAGCGGTTATG |
| JG1519 | ATATGGCCATCTCACTCATTTGATCCATAGGACGTGG |
| JG1520 | GATAAGCTTGATCACGTTTAAACAAGGAGGATAATTAAATACGT |
| JG1553 | AGGAATTCGATATTTGTTTAAACCCATCACCGACACCCG |
| JG1554 | ATAGGCCTGAGATTTGGTGATAGGCTATCTGGTTTAG |
| JG1555 | ATATGGCCATCTCACCTTACTTAGCGAGTATGCCTATG |
| JG1556 | GATAAGCTTGATCACGTTTAAACTGAGGCTCAGTCGTGCT |
| JG1557 | AGGAATTCGATATTTGTTTAAACAAGCCTGGGTGCTTAACA |
| JG1558 | ATAGGCCTGAGATTTTTTCTAATTGATTCTTAGTTAATTGAA |
| JG1559 | ATATGGCCATCTCACCTATTTTTGATATCCCGGTTATAG |
| JG1560 | GATAAGCTTGATCACGTTTAAACATATTATGAATCGGGTGCTC |
| JG1561 | CTCCACCTAAGCCATAACAAGC |
| JG1562 | CCTTCGGCTGTGAAGAGTAGAT |
| JG1563 | GGGAAGGGATTAGAGAGTGTCC |
| JG1564 | TCACAAAGAAACCACCACCT |
| JG1565 | AGGAATTCGATATTTGTTTAAACTCCATACGCAGAAGTCTAA |
| JG1566 | ATAGGCCTGAGATTTCGTCTAGCTGGGGTTCCTGA |
| JG1567 | ATATGGCCATCTCACAAATTAAGACGGCCTTTTTCCTTT |
| JG1568 | GATAAGCTTGATCACGTTTAAACCAGATAGCCTATCACCATG |
| JG1575 | AGGAATTCGATATTTGTTTAAACACGAGCCCTTTATTTGAG |
| JG1576 | ATAGGCCTGAGATTTGATGGATTGTTTGAGGTTTCAGA |
| JG1577 | ATATGGCCATCTCACAGAGCAACGGAGGCTGGGGA |
| JG1578 | GATAAGCTTGATCACGTTTAAACTTGACCCCCTGGGACTT |
| JG1579 | AGGAATTCGATATTTGTTTAAACCGGTTCATGCTGTCCTG |
| JG1580 | ATAGGCCTGAGATTTATAAAGGCGGTTCTGGGAGAAG |
| JG1581 | ATATGGCCATCTCACTTGTCTCACCTGTGGACCTGC |
| JG1582 | GATAAGCTTGATCACGTTTAAACCGTCGTCTCTTTCTACAG |
| JG1690 | AAGTGAAAAGCCACGCTACTG |
| JG1691 | CGGCACAACAACAGAGAGAAT |
| JG1692 | CGATGTTGTTGTGTGTGTGG |
| JG1693 | CCAATCCGTCTTTCATAGTCAA |
| JG1702 | TCTTATGCTATTCCTGGTCCTG |
| JG1703 | GTGGGAGAGAAGTTTGTGGATG |
| JG1704 | CTCGTGGCGTTTCTATGATTC |
| JG1705 | ATCTTTAGGTTGGGTCGTTGAT |

**Supplementary Table 2**: Primers used for construction of plasmids carrying deletion cassettes.

| **Plasmid name** | **Primer for 5’ flanking region** | **Primer for 3’ flanking region** | **Description** |
| --- | --- | --- | --- |
| pME5195 | JG1454/1455 | JG1456/1457 | Deletion of AN7893 |
| pME5231 | JG1575/1576 | JG1577/1578 | Deletion of AN7894 |
| pME5232 | JG1579/1580 | JG1581/1582 | Deletion of AN7895 |
| pME5229 | JG1565/1566 | JG1567/1568 | Deletion of *dbaA* |
| pME5227 | JG1553/1554 | JG1555/1556 | Deletion of *dbaB* |
| pME5228 | JG1557/1558 | JG1559/1560 | Deletion of *dbaC* |
| pME5217 | JG1517/1518 | JG1519/1520 | Deletion of *dbaE* |
| pME5199 | JG1458/1459 | JG1460/1461 | Deletion of *dbaH* |
| pME5189 | JG1437/1438 | JG1439/1440 | Deletion of *dbaI* |

**Supplementary Table 3**: Plasmids constructed and used in this study.

| **Plasmid name** | **Description** | **Reference** |
| --- | --- | --- |
| pME5195 | Deletion of *troA* | This study |
| pME5231 | Deletion of AN7894 | This study |
| pME5232 | Deletion of AN7895 | This study |
| pME5229 | Deletion of *dbaA* | This study |
| pME5227 | Deletion of *dbaB* | This study |
| pME5228 | Deletion of *dbaC* | This study |
| pME5217 | Deletion of *dbaE* | This study |
| pME5199 | Deletion of *dbaH* | This study |
| pME5189 | Deletion of *dbaI* | This study |
| pME4319 | Vector with recyclable phleomycin resistance | (Liu et al., 2021) |
| pBluescript SK(+) | Cloning vector, *amp*^R^ | Thermo Scientific |

**Supplementary Table 4**: *Aspergillus nidulans* strains constructed and used in this study.

| **Name** | **Description** | **Genotype** | **Reference** |
| --- | --- | --- | --- |
| AGB552 | Reference strain (rf) | *pabaA1*; Δ*nkuA*::*argB* | (Bayram et al., 2012) |
| AGB1418 | ΔtroA | Δ*troA*(AN7893)::*β*-*six-site*; *pabaA1*; Δ*nkuA*::*argB* | This study |
| AGB1433 | ΔAN7894 | ΔAN7894::*β*-*six-site*; *pabaA1*; Δ*nkuA*::*argB* | This study |
| AGB1434 | ΔAN7895 | ΔAN7895::*β*-*six-site*; *pabaA1*; Δ*nkuA*::*argB* | This study |
| AGB1447 | Δ*dbaA* | Δ*dbaA*::*β*-*six-site*; *pabaA1*; Δ*nkuA*::*argB* | This study |
| AGB1430 | Δ*dbaB* | Δ*dbaB*::*β*-*six-site*; *pabaA1*; Δ*nkuA*::*argB* | This study |
| AGB1431 | Δ*dbaC* | Δ*dbaC*::*β*-*six-site*; *pabaA1*; Δ*nkuA*::*argB* | This study |
| AGB533 | Δ*dbaD* | Δ*dbaD*::*ptrA; pabaA1*; Δ*nkuA*::*argB* | (Gerke et al., 2012) |
| AGB1432 | Δ*dbaE* | Δ*dbaE*::*β*-*six-site*; *pabaA1*; Δ*nkuA*::*argB* | This study |
| AGB535 | Δ*dbaF* | Δ*dbaF*::*ptrA; pabaA1*; Δ*nkuA*::*argB* | (Gerke et al., 2012) |
| AGB536 | Δ*dbaG* | Δ*dbaG*::*ptrA; pabaA1*; Δ*nkuA*::*argB* | (Gerke et al., 2012) |
| AGB1419 | Δ*dbaH* | Δ*dbaH*::*β*-*six-site*; *pabaA1*; Δ*nkuA*::*argB* | This study |
| AGB1417 | Δ*dbaI* | Δ*dbaI*::*β*-*six-site*; *pabaA1*; Δ*nkuA*::*argB* | This study |
| AGB527 | OE *dbaA* | *ptrA*^R^-*^p^niiA*-*dbaA*; *pyroA4*; *pyrG89*; Δ*nkuA*::*argB*; *veA1* | (Gerke et al., 2012) |

**Supplementary Table 5**: Primers used for real-time PCR.

| **Primer names** | **Gene** |
| --- | --- |
| JG880/881 | *gpdA* |
| JG882/883 | *h2A* |
| JG884/885 | *rps15* |
| JG1704/1705 | AN7890 |
| JG1702/ 1703 | AN7891 |
| JG1690/1691 | AN7892 |
| JG1480/1481 | *troA* |
| JG1561/1562 | AN7894 |
| JG1563/1564 | AN7895 |
| JG680/681 | *dbaA* |
| JG682/683 | *dbaB* |
| JG684/685 | *dbaC* |
| JG686/687 | *dbaD* |
| JG688/689 | *dbaE* |
| JG690/691 | *dbaF* |
| JG692/693 | *dbaG* |
| JG1478/1479 | *dbaH* |
| JG1445/1446 | *dbaI* |
| JG1692/1693 | AN7904 |

**Supplementary Table 6**: Metabolites identified in this study. A = exact mass, B = UV/VIS spectrum, C = MS^2^ spectrum, D = comparison with purified compound (identified by NMR), E = comparison with commercial compound

| **Number** | **Compound name** | **Molecular formula**  **[M]** | **Calc. exact mass of [M]** | **Retention time in min** | **Detected as** | **Measured exact mass** | **Confirmed by** | **Reference** |
| --- | --- | --- | --- | --- | --- | --- | --- | --- |
| 1 | Anhydrosepedonin | C_11_H_10_O_4_ | 206.057910 | 10.35 | [M+H]^+^ | 207.0651 | A, B, C, D | (Divekar et al., 1965; Wright et al., 1970; Quang et al., 2010) |
| 2 | Antibiotic C | C_11_H_10_O_5_ | 222.052824 | 10.60 | [M+H]^+^ | 223.0597 | A, D | (McDonald et al., 1983) |
| 3 | DHMBA | C_11_H_12_O_4_ | 208.073560 | 10.32 | [M+H]^+^ | 209.0806 | A, B, D | (Gerke et al., 2012) |
| 4 | Azanidulone | C_11_H_10_O_4_ | 206.057910 | 6.19 | [M+H]^+^ | 207.0651 | D |  |
| 5 | Tripyrnidone | C_17_H_16_O_7_ | 332.089605 | 7.43 | [M+H]^+^ | 333.0961 | D |  |
| 6 | Sepedonin | C_11_H_12_O_5_ | 224.068475 | 6.6 | [M+H]^+^ | 225.0756 | A, B | (Supka, 1981) |
| 7 | Triacetic acid lactone (TAL) | C_6_H_6_O_3_ | 126.031695 | 5.35 | [M+H]^+^ | 127.0390 | A, E |  |

**Supplementary Table 7**: NMR data (^1^H 500 MHz, ^13^C 125 MHz) of anhydrosepedonin (**1**) in
DMSO-*d*_6_

| **Atom#** | **δ_C_, mult.** | **δ_H_, mult.** | **NOESY** | **C to H HMBC** |
| --- | --- | --- | --- | --- |
| 1 | 169.8, C |  |  |  |
| 2 | 165.8, C |  |  |  |
| 3 | 112.4, CH | 6.59, s | 8 | 11, 8, 5, 4, 6, 2, 1 |
| 4 | 140.9, C |  |  |  |
| 5 | 112.7, C |  |  |  |
| 6 | 161.6, C |  |  |  |
| 7 | 111.2, CH | 6.76, s |  | 11, 5, 4, 6, 2, 1 |
| 8 | 105.2, CH | 5.73, s | 10, 3 | 10, 11, 3, 4, 9 |
| 9 | 161.2, C |  |  |  |
| 10 | 19.1, CH_3_ | 1.93, s | 8 | 8, 5, 4, 9 |
| 11 | 64.5, CH_2_ | 5, s |  | 8, 7, 5, 4, 9 |

**Supplementary Table 8**: NMR data (^1^H 500 MHz, ^13^C 125 MHz) of antibiotic C (**2**) in
DMSO-*d*_6_

| **Atom#** | **δ_C_, mult.** | **δ_H_, mult.** | **NOESY** | **C to H HMBC** |
| --- | --- | --- | --- | --- |
| 1 | 154.3, C |  |  |  |
| 2 | 146.5, C |  |  |  |
| 3 | 113.2, CH | 6.59, s | 8 | 11, 8, 5, 4, 2, 1 |
| 4 | 132.9, C |  |  |  |
| 5 | 114.1, C |  |  |  |
| 6 | 155.1, C |  |  |  |
| 7 | 160.9, C |  |  |  |
| 8 | 104.2, CH | 5.70, s | 10, 3 | 10, 3, 5, 4, 6, 9 |
| 9 | 157.8, C |  |  |  |
| 10 | 19.1, CH_3_ | 1.91, s | 8 | 8, 9 |
| 11 | 64.6, CH_2_ | 5.14, s |  | 5, 4, 6, 9, 7 |

**Supplementary Table 9: List of species with detected *dba/troA* gene cluster.** The JGI fungal genome database (https://mycocosm.jgi.doe.gov/mycocosm/home) was accessed on Nov. 16^th^, 2021 for blast analysis of all deposited genomes including 267 *Aspergillus* species which were queried with the DNA sequence of *A. nidulans* *dba/troA* gene cluster.

| **Genus** | **Species** | **Strain** |
| --- | --- | --- |
| *Aspergillus* | *Aspergillus acristatulus* | CBS 119.55 |
|  | *Aspergillus aurantiopurpureus* |  |
|  | *Aspergillus cleistominutus* | CBS 200.75 |
|  | *Aspergillus conjunctus* | CBS 476.65 |
|  | *Aspergillus dentatus* | CBS 114.63 |
|  | *Aspergillus desertorum* | CBS 653.73 |
|  | *Aspergillus falconensis* | CBS 271.91 |
|  | *Aspergillus foveolata* | CBS 279.81 |
|  | *Aspergillus fructiculosus* |  |
|  | *Aspergillus granulosus* | CBS 588.65 |
|  | *Aspergillus indicus* |  |
|  | *Aspergillus navahoensis* |  |
|  | *Aspergillus neoechinulatus* | CBS120.55 |
|  | *Aspergillus nidulans* |  |
|  | *Aspergillus quadrilineatus (floriformis)* | CBS 937.73 |
|  | *Aspergillus quadrilineatus (gemmatus)* | CBS 853.96 |
|  | *Aspergillus similis* |  |
|  | *Aspergillus sublatus* | IBT 19356 |
|  | *Aspergillus tanneri* | DTO 303-18 |
|  | *Aspergillus tetrazonus* | CBS 591.65A |
| *Talaromyces* | *Talaromyces stipitatus* | ATCC 10500 |
| *Coccidioides* | *Coccidioides immitis* | RS |
|  | *Coccidioides posadasii* | C735 delta SOWgp |
|  | *Coccidioides posadasii* | *Silveira* |

^1^H NMR spectrum (500 MHz, DMSO-*d*_6_) of anhydrosepedonin (**1**).

^13^C NMR spectrum (125 MHz, DMSO-*d*_6_) of anhydrosepedonin (**1**).

COSY NMR spectrum (500 MHz, DMSO-*d*_6_) of anhydrosepedonin (**1**).

NOESY NMR spectrum (500 MHz, DMSO-*d*_6_) of anhydrosepedonin (**1**).

HSQC NMR spectrum (500 MHz, DMSO-*d*_6_) of anhydrosepedonin (**1**).

HMBC NMR spectrum (500 MHz, DMSO-*d*_6_) of anhydrosepedonin (**1**).

^1^H NMR spectrum (500 MHz, DMSO-*d*_6_) of antibiotic C (**2**).

^13^C NMR spectrum (125 MHz, DMSO-*d*_6_) of antibiotic C (**2**).

NOESY NMR spectrum (500 MHz, DMSO-*d*_6_) of antibiotic C (**2**).

HSQC NMR spectrum (500 MHz, DMSO-*d*_6_) of antibiotic C (**2**).

HMBC NMR spectrum (500 MHz, DMSO-*d*_6_) of antibiotic C (**2**).

^1^H NMR spectrum (500 MHz, DMSO-*d*_6_) of tripyrnidone (**5**).

^13^C NMR spectrum (125 MHz, DMSO-*d*_6_) of tripyrnidone (**5**).


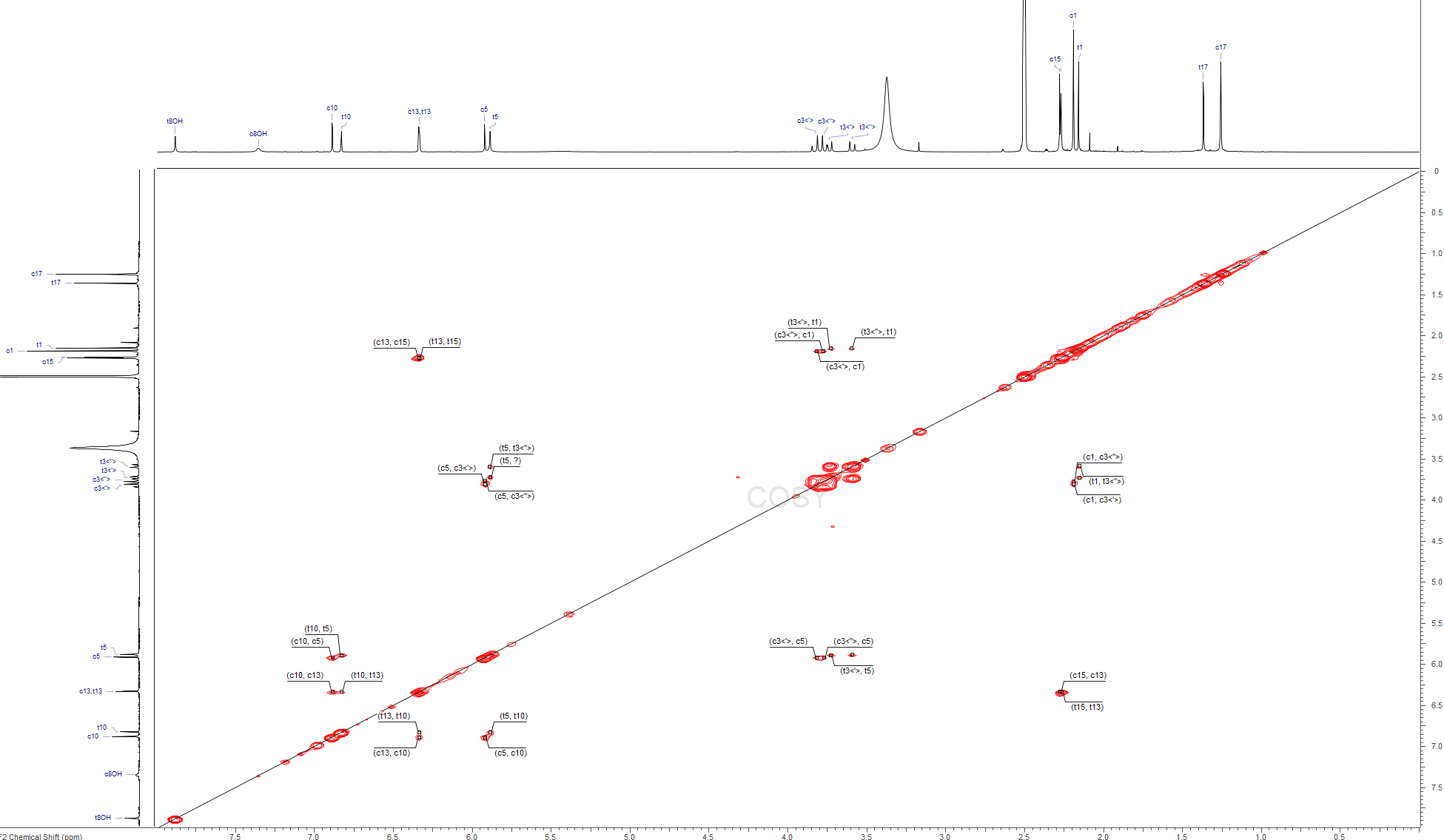


COSY NMR spectrum (500 MHz, DMSO-*d*_6_) of tripyrnidone (**5**).


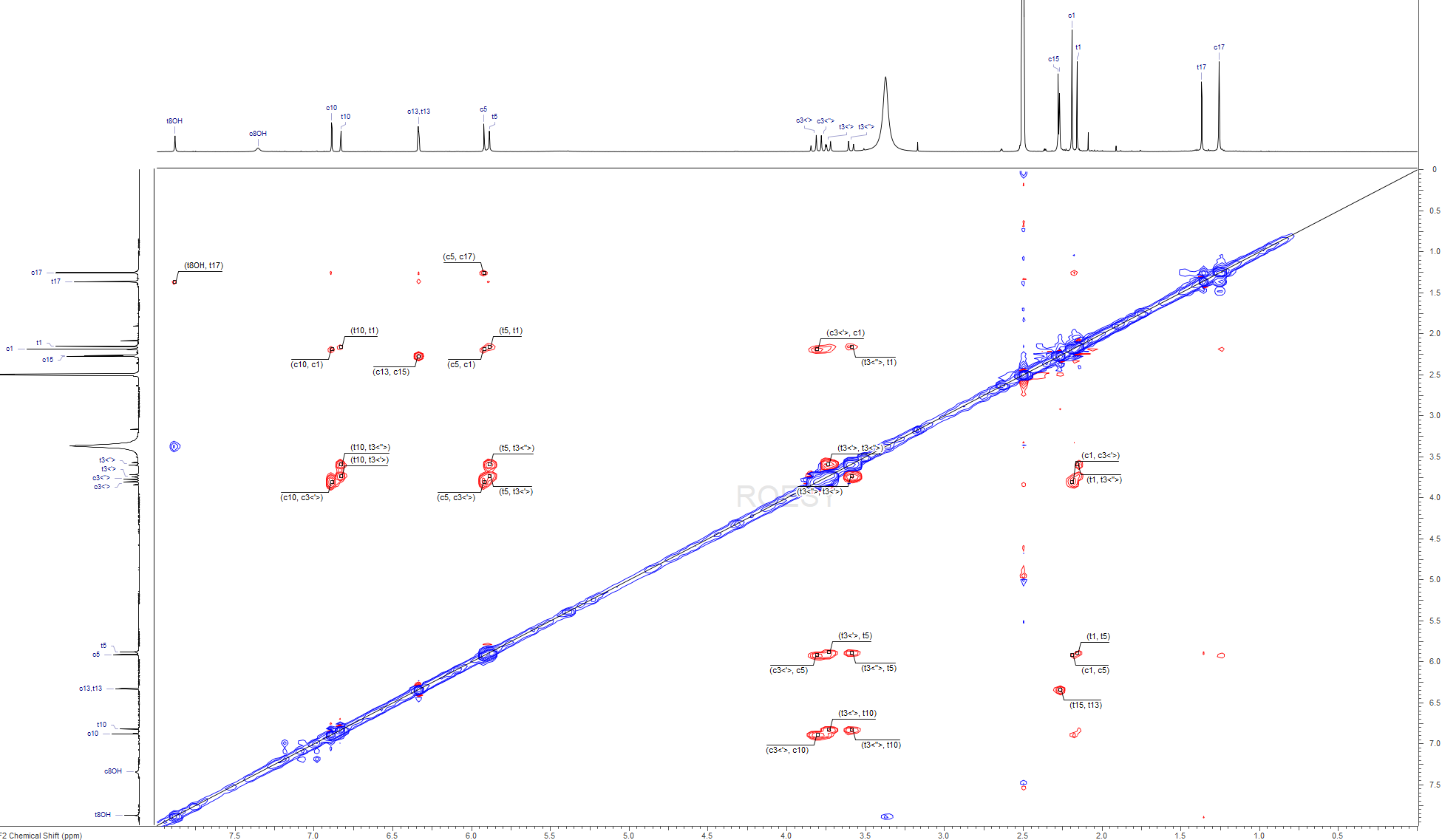


ROESY NMR spectrum (500 MHz, DMSO-*d*_6_) of tripyrnidone (**5**).


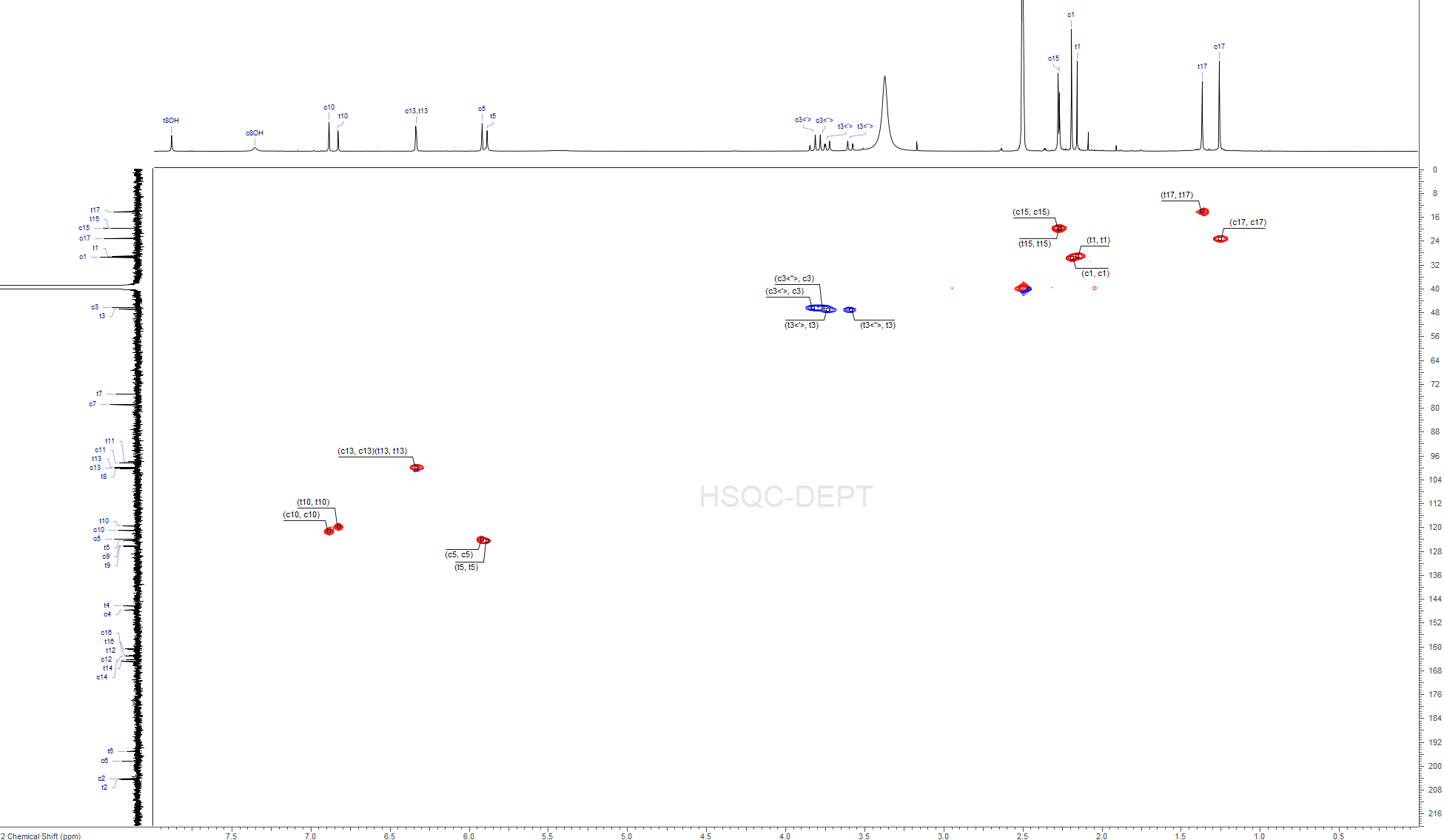


HSQC NMR spectrum (500 MHz, DMSO-*d*_6_) of tripyrnidone (**5**).


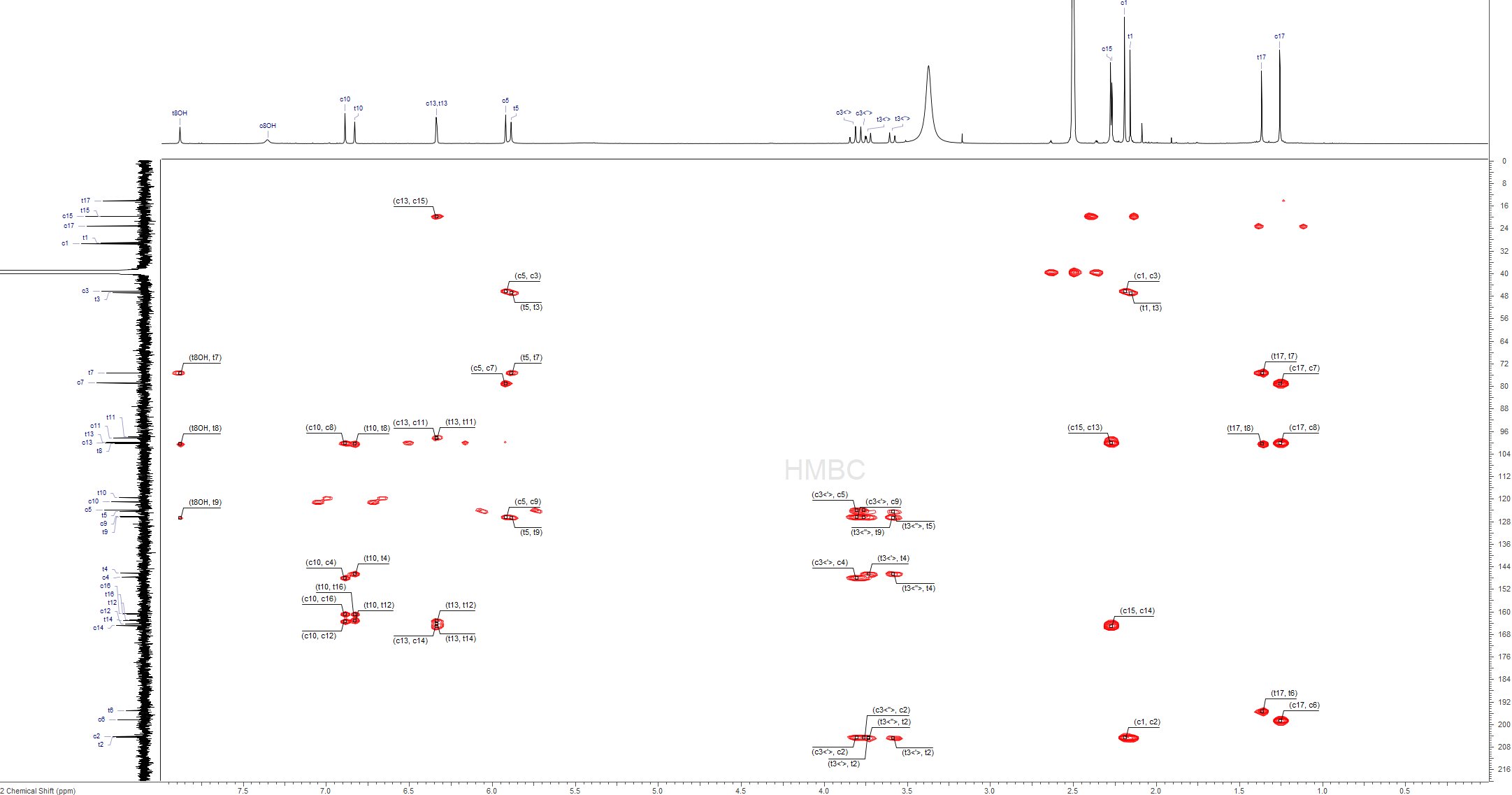


HMBC NMR spectrum (500 MHz, DMSO-*d*_6_) of tripyrnidone (**5**).


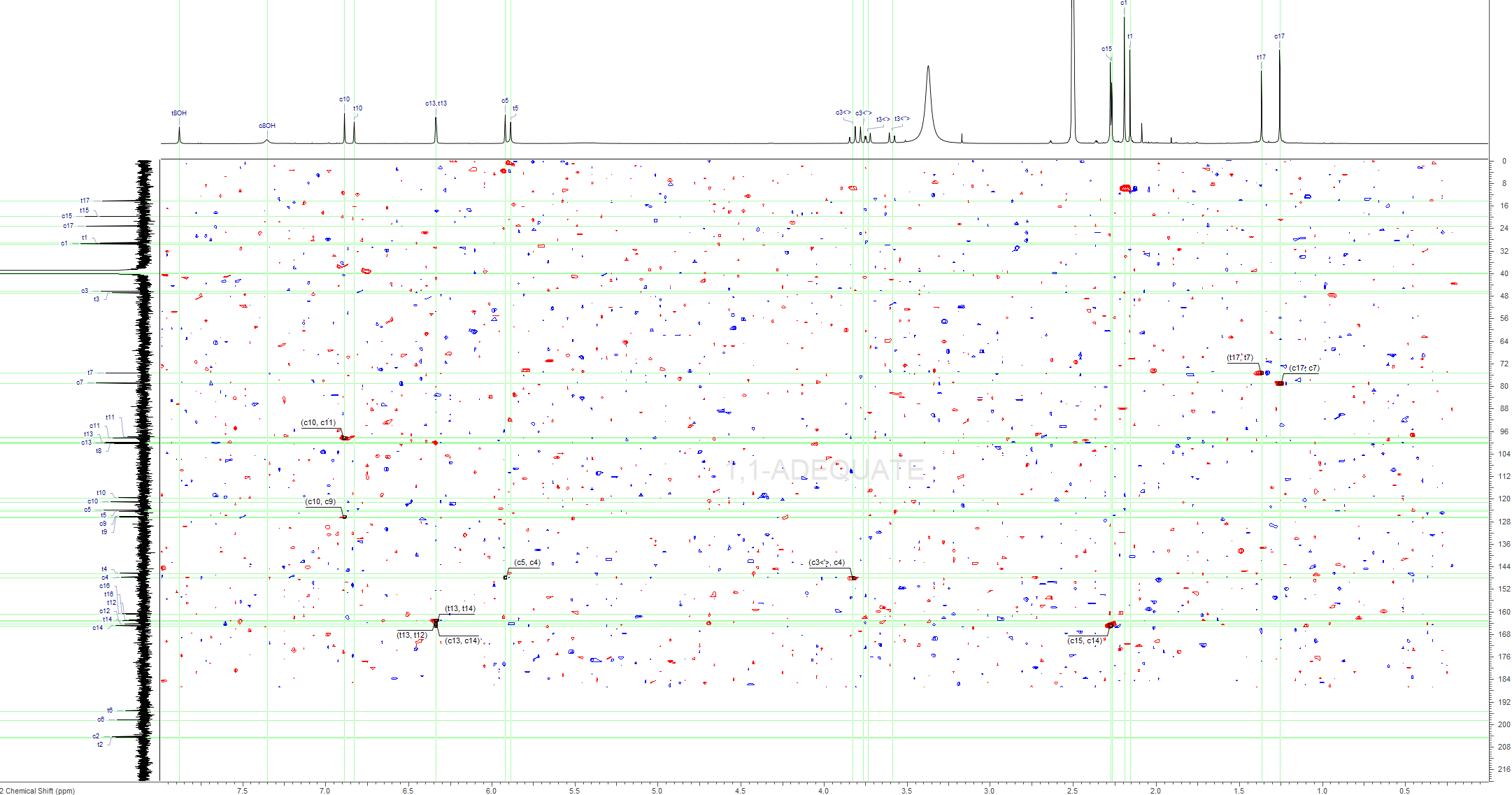


1,1-ADEQUATE NMR spectrum (700 MHz, DMSO-*d*_6_) of tripyrnidone (**5**).

**References**

Bayram, Ö., Bayram, Ö. S., Ahmed, Y. L., Maruyama, J., Valerius, O., Rizzoli, S. O., et al. (2012). The *Aspergillus nidulans* MAPK Module AnSte11-Ste50-Ste7-Fus3 Controls Development and Secondary Metabolism. *PLoS Genet.* 8, e1002816. doi:10.1371/journal.pgen.1002816.

Davison, J., Al Fahad, A., Cai, M., Song, Z., Yehia, S. Y., Lazarus, C. M., et al. (2012). Genetic, molecular, and biochemical basis of fungal tropolone biosynthesis. *Proc. Natl. Acad. Sci. U. S. A.* 109, 7642–7647. doi:10.1073/pnas.1201469109.

Divekar, P. V., Raistrick, H., Dobson, T. A., and Vlning, L. C. (1965). Studies in the biochemistry of microorganisms part 117.1 Sepedonin, a tropolone metabolite of *Sepedonium chrysospermum* Fries. *Can. J. Chem.* 43, 1835–1848. doi:10.1139/v65-241.

Gerke, J., Bayram, Ö., Feussner, K., Landesfeind, M., Shelest, E., Feussner, I., et al. (2012). Breaking the Silence: Protein Stabilization Uncovers Silenced Biosynthetic Gene Clusters in the Fungus *Aspergillus nidulans*. *Appl. Environ. Microbiol.* 78, 8234–8244. doi:10.1128/AEM.01808-12.

Grigoriev, I. V, Nikitin, R., Haridas, S., Kuo, A., Ohm, R., Otillar, R., et al. (2014). MycoCosm portal: gearing up for 1000 fungal genomes. *Nucleic Acids Res.* 42, D699-704. doi:10.1093/nar/gkt1183.

Liu, L., Sasse, C., Dirnberger, B., Valerius, O., Fekete-Szücs, E., Harting, R., et al. (2021). Secondary metabolites of Hülle cells mediate protection of fungal reproductive and overwintering structures against fungivorous animals. *Elife* 10. doi:10.7554/eLife.68058.

McDonald, K. D., Middleton, A. J., and Cole, D. S. (1983). Antibiotic. UK Patent Application GB 2 113 672 A. The Patent Office London, U.K.

Quang, D. N., Schmidt, J., Porzel, A., Wessjohann, L., Haid, M., and Arnold, N. (2010). Ampullosine, a new isoquinoline alkaloid from *Sepedonium ampullosporum* (Ascomycetes). *Nat. Prod. Commun.* 5, 869–872. doi:10.1177/1934578x1000500609.

Sharpton, T. J., Stajich, J. E., Rounsley, S. D., Gardner, M. J., Wortman, J. R., Jordar, V. S., et al. (2009). Comparative genomic analyses of the human fungal pathogens Coccidioides and their relatives. *Genome Res.* 19, 1722–1731. doi:10.1101/gr.087551.108.

Supka, R. (1981). Sepedonin und Anhydrospedonin - Zwei Tropolon-Antibiotika aus Sepedonium chrysospermum Bulliard Ex Fries. [PhD thesis]. [Tübingen, Germany]; Eberhard-Karls-Universität Tübingen.

Wright, J. L. C., McInnes, A. G., Smith, D. G., and Vining, L. C. (1970). Structure of sepedonin, a tropolone metabolite of *Sepedonium chrysospermum* Fries. *Can. J. Chem.* 48, 2702–2708. doi:10.1139/v70-456.
